# Access to information in deaf and hard-of-hearing people

**DOI:** 10.1371/journal.pone.0343904

**Published:** 2026-03-02

**Authors:** Eva Gutierrez-Sigut, Veronica Lamarche, Katherine Rowley, Emilio Ferreiro Lago, María Jesús Pardo-Guijarro, Ixone Saenz, Berta Frigola, Santiago Frigola, Delfina Aliaga, Laura Goldberg

**Affiliations:** 1 Department of Psychology, University of Essex, Colchester, United Kingdom; 2 DCAL Research Centre, University College London, London, United Kingdom; 3 University of Castilla-La Mancha, Cuenca, Spain; 4 University Pompeu Fabra Barcelona, Spain; 5 Barcelona, Spain; University of Padova, ITALY

## Abstract

Deaf and Hard of Hearing (HoH) people often face communication barriers that limit their access to crucial information. This study investigates which modalities deaf people used to gather information from various sources, and which factors predicted accessibility, satisfaction, and wellbeing. We report the findings from an accessible survey study, released in two written and three signed languages. Responses from 395 deaf/HoH UK and Spanish residents were collected online shortly after the COVID-19 pandemic breakout. We investigated whether the onset and Level of Deafness, knowledge of sign language, Residence, and self-assessed reading skill predicted the frequency with which they found accessible information, their satisfaction levels with the information accessed, and their physical and mental health. We found that most deaf/HoH people relied on subtitles to access information from the government and the news, but when signed information was available (i.e., from deaf organisations), SL was the preferred modality of access. Our main finding was that reading skill was a key predictor, with less skilled readers being at a disadvantage, less satisfied, and having lower health outcomes. Our findings advocate for facilitating more signed information, as well as releasing written information at appropriate reading levels and improving literacy programmes for deaf people.

## Introduction

During the COVID-19 pandemic, people’s ability to access reliable and accurate information was crucial for their health and wellbeing. As a consequence, the public sought information more frequently than ever before. This increased demand for information created a unique context in which to study the communication barriers faced by deaf people. Across the globe, important health and safety information was provided mainly through news reports and government updates, interactions with people wearing masks, and written materials. In a study run alongside the current one, we focused on the communication difficulties experienced by deaf people due to face-masks wearing [1]. Here, we investigate which modalities deaf people used to gather information from various sources. We also identify the factors that predicted the frequency with which they found accessible information, their satisfaction levels with the information accessed, and their wellbeing. The present study is important because it reflects communication challenges that societies regularly present to deaf people, not just during the pandemic. These challenges have been linked to lower mental health and quality of life in the deaf population (e.g., [2]) and should be addressed in order to decrease communication barriers for deaf members of society.

The World Health Organization estimates that about 430 million people worldwide have hearing loss [3], including 12 million in the UK [4] and over one million in Spain [5]. The deaf and hard-of-hearing (deaf/HoH) population is extremely diverse in terms of the onset and degree of their deafness, as well as their knowledge of a signed language. For example, in the UK, about 28% of deaf/HoH people were born deaf or became deaf in early childhood, while about 71% became deaf later in life, mostly due to ageing [6]. People with different onset of hearing loss may use different communication strategies [7]. For instance, people with late-onset hearing loss may tend to hide their hearing loss and avoid social interactions [8]. At the same time, they might have had more chances of developing a high reading skill early in childhood and hence prefer written communication. On the other hand, people with early-onset deafness may have learnt sign language (SL) or have developed more effective visual communication strategies speechreading, cued speech, pointing, and gesturing [9,10]. The Level of Deafness could also influence preferences of accessing information. For example, people with severe or profound deafness may not hear speech at all without hearing aids [11,12]. In contrast, people with mild or moderate deafness (HoH) are likely to perceive speech, especially with technological aids (see, e.g., [11] for details on accessible sounds depending on Level of Deafness). In our previous study [1] we found that all of these predictors contributed differently. Communication with people who wore masks was difficult for all participants in all situations, resulting in significant loss of information and increased feeling of disconnection from society. However, it was clear that there were crucial differences in access to information and wellbeing as a function of the degree and onset of hearing loss, Knowledge of SL, and cultural context (i.e., Residence: Spain vs. UK in our sample). Specifically, late onset deafness was associated with more communication difficulties and lower wellbeing. At the same time, severely or profoundly deaf people, regardless of the onset reported higher loss of information and more disconnection from society. Signing status and Residence (both having a facilitatory effect) explained differences in difficulties experienced in social communication and the degree of use of other types of visual communication (gesturing, writing, etc.). In the present work we used these predictors as they have proven useful to capture the inherent variability within the deaf communities as well as the surrounding social context. James et al. [13] found that people who are deaf and HoH with low health literacy remained at risk of information marginalization during the pandemic, independently of their language preference. Conversely, Almusawi et al. [14] found that in Saudi Arabia the Level of Deafness and use of sign language as the primary means of communication were associated with lower health knowledge scores. The authors also found that deaf/HoH participants relied on social media more than hearing people, who mainly used official government sources. Differences in media accessibility policies between countries likely influenced deaf people’s access to information during the pandemic. For example, the UK’s British Sign Language Act mandates BSL use in government communications but does not set broadcast quotas [15], whereas Spain’s Audiovisual Communication Law requires 90% captioning and at least 15 hours of sign-language interpretation weekly on public television [16]. During COVID-19, the lack of BSL in UK government briefings was widely criticised [17], while Spain maintained regulated accessibility standards for broadcast content. Finally, given the high value of reading to access information in western societies, here we also include self-assessed Reading Skill as a predictor. Below we focus on the importance of reading skill.

A great amount of information necessary to keep safe was distributed in writing during COVID-19 pandemic. This was certainly useful for many people, as captured by a surge in reading behaviours during the COVID-19 pandemic [18,19]. Salmerón et al. [19] observed a significant increase of news reading, as well as reading to socialize and to work in the general population, particularly at initial stages of the pandemic. Having to depend on written materials likely put many deaf people at disadvantage because literacy levels are typically lower in the deaf population in comparison to the general population due to not having enough access to language (signed or spoken) in the early years of life (0–5 years) [20]. It is well-known that many deaf people leave school with, on average, a reading age of about 8–11 years (e.g., [21,22] or lower [23]. It is important to note that some deaf people do become fluent readers, often because they have been able to access language at an early age and have had access to the language of instruction (whether that is through spoken language through the aid of hearing technologies -if they have sufficient hearing- or sign language). Simple readability checks of a few random texts being distributed in April 2020, containing essential information to stop the spread of the virus at public places such as shops, raised a red flag in terms of accessibility. We found several public weekly updates to have a Flesch Reading ease score [24] of 43.8, which corresponds to college-level difficulty. This observation was in line with previous reports that all materials developed to prepare deaf/HoH readers for emergencies were above the recommended reading level [25], indicating that important health information is likely not accessible for vulnerable populations (both deaf and hearing, [26,27]. In addition to potentially inaccessible written materials from different sources, there are other communication barriers that reflect society’s lack of understanding of the communication needs of deaf people. For example, if the televised news and updates were not accompanied by SL interpretation, many deaf people only had access through reading subtitles, which requires a certain level of reading proficiency. Furthermore, if medical practitioners do not use SL and communication difficulty is increased due to face-masks, they tend to write their messages [28], but the efficacy of these messages heavily depends on the reading skill of the deaf message recipient. In sum, accessibility for deaf/HoH people is shaped by broad systemic issues, especially regarding the availability of sign language interpreters and accessible resources. The most important are insufficient interpreter access, and over-reliance on written or spoken language, which often excludes those who primarily use sign language [29–36]. In the present study we investigate accessibility of information, as well as satisfaction with the information accessed, and health, in a large population of deaf/HoH people across two languages.

Importantly, in order to conduct research that represents well the deaf/HoH populations, we provided participants with the opportunity to see the survey in their natural language. Therefore, we released our survey not only on two written languages but also three different sign languages [British Sign Language (BSL), Spanish Sign Language (Lengua de Signos Española: LSE) and Catalan Sign Language (Llengua de Signes Catalana: LSC)].

The present study

**Fig 1:**
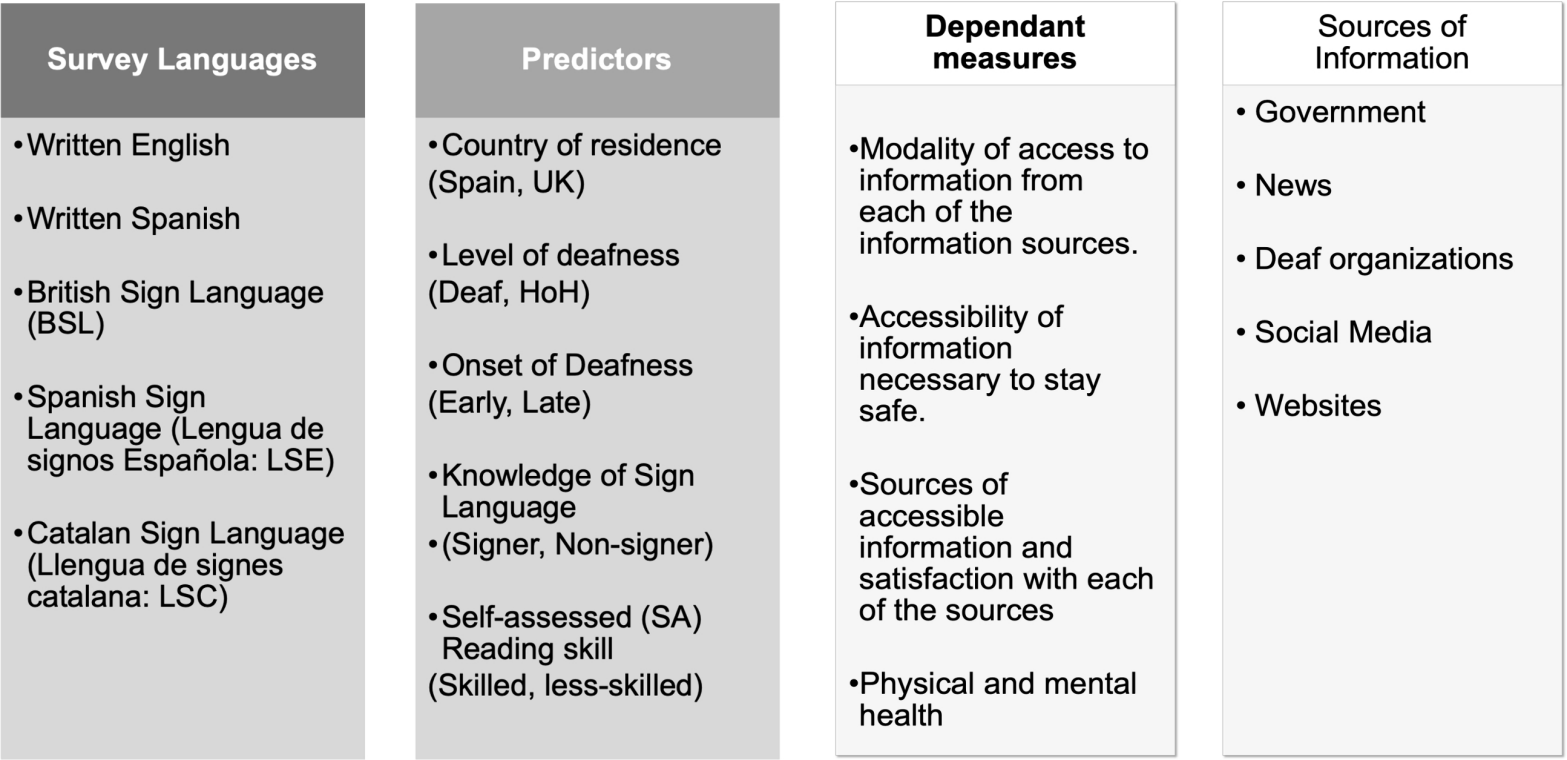
Summary of the present study.

Fig 1 shows a summary of the modalities of access, sources of information and predictor variables used in this study. The primary aims of the study were to:

a) Describe which modalities Deaf/HoH people used to access important information from both official (e.g., televised government updates) and nonofficial (e.g., social media) sources. We were particularly interested in exploring deaf people’s access to information provided by deaf organisations as they were the only sources to provide a constant stream of signed information.
b) Analize how often Deaf/HoH people felt they had access to reliable information to stay safe across various modalities, as well as how satisfied they were with that information.
c) Assess how predictors such as level and Onset of Deafness, Knowledge of SL, Reading Skill, and Residence influenced access to information and wellbeing. Note that these variables have been identified in previous studies as key factors influencing accessibility and wellbeing within the deaf population (e.g., [37].

## Methods

### Participants and materials

Deaf people who participated in the Gutierrez-Sigut et al. [1] study was offered the option to also answer the questions reported here. All participants from our previous study [1] chose to answer the present questions about their access to information since the COVID-19 outbreak, therefore participants are the same. Only complete datasets from deaf/HoH people living in Spain and the UK were downloaded from the Qualtrics platform and analysed (an additional 109 datasets did not have answers to any question because the participants did not click past the information sheet to access the consent form nor the survey questions). A total of 395 deaf/HoH people from the UK (n = 273) and Spain (n = 122) answered these questions voluntarily in exchange for participation one of six online retailer vouchers (£50 or 50€ depending on their country of residency). Twenty eight percent of people (112 respondents) completed the sign language version of the survey. There were no obvious differences in gender, age or Residence between the participants that accessed the SL and written version. As expected, respondents of the SL version were mostly severely or profoundly deaf (96%), with early onset deafness (96%), and were signers. Respondents who chose the written version were more equally distributed in terms of Knowledge of SL (45% non-signers), Onset of Deafness (42% early) and Level of Deafness (26% HoH). Participants who chose the written version were mostly skilled readers (80%) while reading skill was more balanced amongst those who chose the SL version (52% less skilled readers).

On average participants were 45.6 years old (SD = 15.9, range 18−81; see characteristics in Table 1). Participants were recruited through word of mouth, social media posts, and deaf organisations email distribution lists. This study was approved by University of Essex Science and Health Ethics Sub-committee (ETH2021−0196).

**Table 1:** Participants’ characteristics. All percentages are the values in relation to the total sample.

	UK	Spain	Total
	% (N)	*% (N)*	*% (N)*
**Gender**			
Female	49.4% (N = 195)	19.7% (N = 78)	69.1% (N = 273)
Male	18.7% (N = 74)	9.9% (N = 39)	28.6% (N = 113)
Genderqueer		0.5% (N = 2)	0.5% (N = 2)
Transgender	0.3% (N = 1)		0.3% (N = 1)
Prefer not to say	0.8% (N = 3)	0.8% (N = 3)	1.5% (N = 6)
**Level of deafness**			
Hard of hearing	20% (N = 79)	7.1% (N = 28)	27% (N = 107)
Deaf	49.1% (N = 194)	23.8% (N = 94)	73% (N = 288)
**Onset of deafness**			
Early	45.3% (N = 179)	23.8% (N = 94)	69.1% (N = 273)
Late	24.3% (N = 96)	6.6% (N = 26)	30.9% (N = 122)
**Knowledge of SL**			
Signer	50.1% (N = 198)	22% (N = 87)	72.2% (N = 285)
Non-signer	18.7% (N = 74)	8.9% (N = 35)	27.8% (N = 109)
**Self-assessed reading skill**			
Skilled	78.3% (N = 213)	56.6% (N = 69)	71.6% (N = 282)
Less skilled	21.7% (N = 59)	43.4% (N = 53)	28.4% (N = 112)
**Total**	68.9% (N = 273)	30.8% (N = 122)	

### Predictor variables

Residence, Level of Deafness, Onset of Deafness and Knowledge of SL were defined, and vector coded as in Gutierrez-Sigut et al. (2022). **Residence**: −1 = Spain and 1 = UK. **Knowledge of SL:** −1 = non-signer and 1 = signer. **Level of Deafness:** −1 = HoH and 1 = deaf. We classified respondents with mild or moderate hearing loss in their best ear as HoH, and those with severe or profound loss as deaf. HoH people have some access to speech sounds without aids, while severely or profoundly deaf people generally have very little or no access to speech sounds without aids. **Onset of Deafness:** −1 = Late and 1 = early. Onset People that became deaf at age 10 or older were considered late-onset, while people becoming deaf before the age of 9 years old were considered early-onset. This distinction was based on previous studies with deaf people who considered age 9 as the cut-off age for early language development (for a recent review see [38].

**Self-assessed (SA) Reading Skill** was measured through responses to the question “*Do you consider yourself a good reader?*”. The response options [1–5] were “1 = *Not good at all*”, “2 = *Poor*”, “3 = *Average*”, “4 = *Good*”, “5 = *Excellent*”. If their answer was “Not good at all”, “Poor” or “Average” participants were considered (−1) less-skilled readers. Conversely, if their answer was “Good” or “Excellent” they were considered [1] skilled readers.

Note that we decided to use self-assessed Reading Skill rather than measuring reading ability with a reading test for several reasons. First, there is evidence that self-assessment of reading skill can be considered a good indicator of actual reading performance (see, e.g., [39,40]. In a review of 57 studies, Falchikov and Boud [39] concluded that student’s self-assessments tend to reflect teacher assessments well. Similar conclusions have been reached in studies of second language learners, where learners were accurate self-assessing their foreign language reading abilities (e.g., [41]. Second, most reading tests are not well adapted to measure reading skill accurately in deaf people [42]. Moreover, the few accessible ones were not readily available for online administration. Third, most tests of reading ability take a long time to complete. Even shorter tests like the Vernon Warden [43] take around ten minutes. Given that the questions for this study were already additional to our previously published research, we considered that it would have taken too long to answer and many of the participants would have chosen to not complete the survey. What is more, those potentially more vulnerable (i.e., deaf people with a lower reading ability) would have been less likely to not complete the survey if it included a long and challenging reading test.

Participants also rated their reading enjoyment (“*How much do you enjoy reading?”* With the responses being “1 = *Not at all*”, “2 = *A little*”, “3 = *A moderate amount*”, “4 = *A lot*”, “5 = *Very much*”), and reported their reading habits (“*Approximately how many books have you read in the past year?”* With the responses being “1 = *0*”, “2 = *1-4*”, “3 = *5-9*”, “4 = *10-14*”, “5 = *15 or more*”). SA Reading Skill was strongly correlated with reading enjoyment (R = 0.606, *p* < .001) and moderately correlated with number of books read (R = 0.495, *p* < .001). These positive correlations are consistent with previous research and validate the use of SA Reading Skill as a proxy to reading ability (see [44–50] for the relationships between reading ability and self-assessed reading skill or self-reported reading habits).

**Fig 2:**
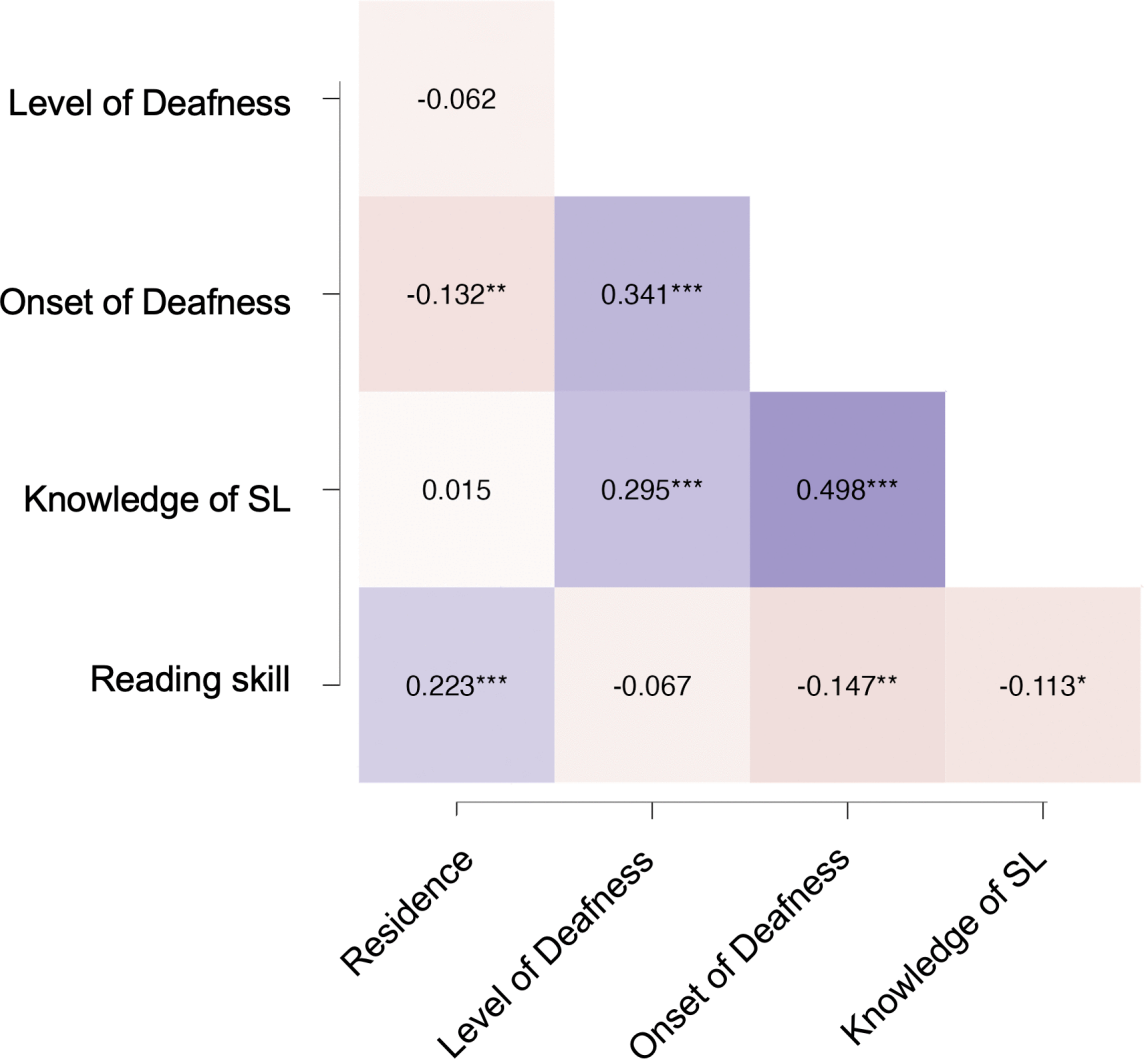
Heatmap showing the correlations between the predictor measures. Darker red colours represent a larger negative correlation while darker purple show a larger positive correlation.

The correlations between our predictors are shown in Fig 2. Weak to moderate significant correlations (all Rs between 0.015 and 0.498) can be observed between our predictors, indicating some degree of shared variance. For example, respondents with early-onset deafness tended to also be signers as well as deaf (as opposed of HoH). Deaf respondents also tended to be signers (as opposed to not knowing SL). To rule out potential multicollinearity, we analised the variance inflation factor (VIF). The VIF for our predictors was between 3.1 and 5.3, suggesting only moderate collinearity that is typically considered manageable, especially if the variables are theoretically important as is the present case.

### Dependent variables

**Modality of access to information.** Participants answered the question “On each of the following sources of information about COVID-19, which ways have you found most of the information?

The sources of information were Official: Government / Official: News / Official: Deaf organisations / Unofficial: Social media / Unofficial: Websites”.

The response options [1–5] were, “1 = spoken language (including lipreading)”, “2 = Sign Language”, “3 = Written: subtitles”, “4 = Written: text”, and “5 = None of these modalities”.

**Accessibility of information necessary to stay safe.** Participants answered the following three questions: 1) “Since the COVID-19 outbreak how much of the time have you felt that you had access to enough information to stay safe”, 2) “Since de COVID-19 outbreak how much of the time have you felt that you had access to enough reliable information from the government to stay safe”, and 3) “Since the COVID-19 outbreak how much of the time have you felt that you had access to enough reliable information from non-governmental sources to stay safe”. For the three questions the response options [1–6] were, “1 = none of the time”, “2 = a little of the time”, “3 = some of the time”, “4 = a good bit of the time”, “5 = most of the time”, and “6 = all of the time”.

#### Sources of accessible information and satisfaction levels.

Regarding frequency of use of the information sources: Participants answered the question “*Since the COVID-19 outbreak I have found accessible information through… Televised daily government updates / Written government information (webpage, leaflets, etc) / Newspapers articles / Deaf organisations official updates / Family or friends / Unofficial: Social media / Unofficial: Websites / Other sources*” the response options [1–6] were, “1 = *none of the time”, “2 = a little of the time”, “3 = some of the time”, “4 = a good bit of the time”*, “5 = *most of the time”, and* “6 = *all of the time”*.

Regarding satisfaction with information sources. Participants answered the question “*How satisfied are you with the information received from… Televised daily government updates / Written government information (webpage, leaflets, etc) / Newspapers articles / Deaf organisations official updates / Family or friends / Unofficial: Social media / Unofficial: Websites / Other sources*” the response options [1–5] were, “1 = *Extremely dissatisfied”, “2 = Somewhat dissatisfied”, “3 = Neither satisfied or dissatisfied”, “4 = Moderately satisfied”*, and “5 = *Extremely satisfied”*.

While adding multiple frequency questions for accessibility and satisfaction lengthened the questionnaire, we considered it important to cover both aspects. Participants might frequently find accessible information from a source but still be dissatisfied with it or might be satisfied with information from a source even if it was not accessible frequently.

**Physical and mental health.** As in Gutierrez-Sigut et al. [1], we used the SF-12 questionnaire v.1 [51] with our own translation to British Sign Language (BSL), Spanish Sign Language (Lengua de Signos Española: LSE) and to Catalan Sign Language (Llengua de Signes |Catalana: LSC) (for details [1]). Note that this questionnaire has not been validated with deaf/HoH populations, therefore we are cautious regarding comparing deaf/HoH people scores to the hearing population and we avoid clinical or diagnostic interpretations of these scores. Accordingly, all results reported here should be understood as associations of the predictors with SF-12 scores within this D/HoH sample. Since the goal of this study is understanding the role of the different predictors on health, we use the raw scores in analyses.

Participants also answered to more specific questions about their mental health. In particular they saw the following questions: “*Since the COVID-19 outbreak how much of the time have you felt …. “ a)* “*more downhearted and blue than before“, b)* “*less calm and peaceful than before, c)* “*more nervous, anxious or on edge than before, d)* “*more lonely than before, e)* “*more worried than before, f)* “that you had *more trouble concentrating on things, such as watching television, than before”, and g)* “that you had *more trouble than before falling asleep of that you sleep too much”.* The response options [1–6] were, “1 = none of the time”, “2 = a little of the time”, “3 = some of the time”, “4 = a good bit of the time”, “5 = most of the time”, and “6 = all of the time”. Note that rather than providing diagnostic insight, we aimed to capture trends in mental health across various aspects that are known to contribute to overall mental well-being.

### Procedure

Participants gave written informed consent via several ticks on a Qualtrics survey, the survey was not shown if participants did not consent to all statements. Participants then answered the survey items online, using their own devices and at their own pace (within a week of the start). Data was collected between the 3^rd^ of November 2020 and the 10^th^ of February 2021, at the same time than the data reported in [1].

## Results

**Fig 3:**
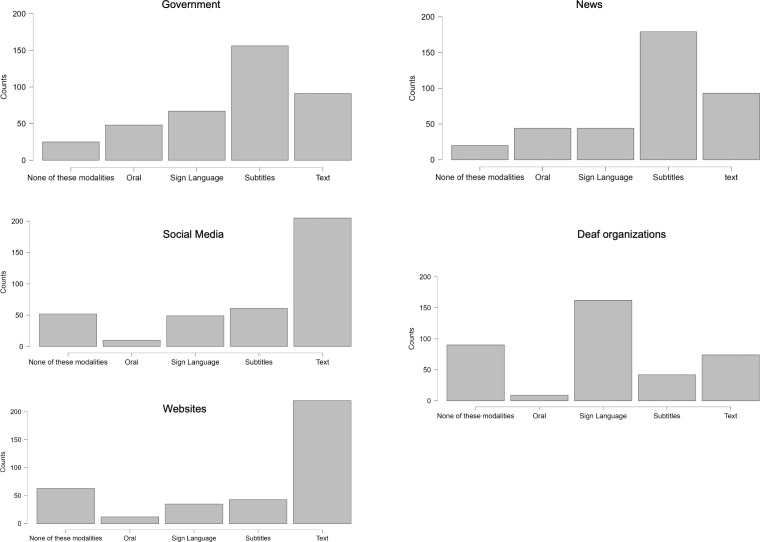
Number of deaf people using each of the modalities for each of the different sources of information.

**Fig 4:**
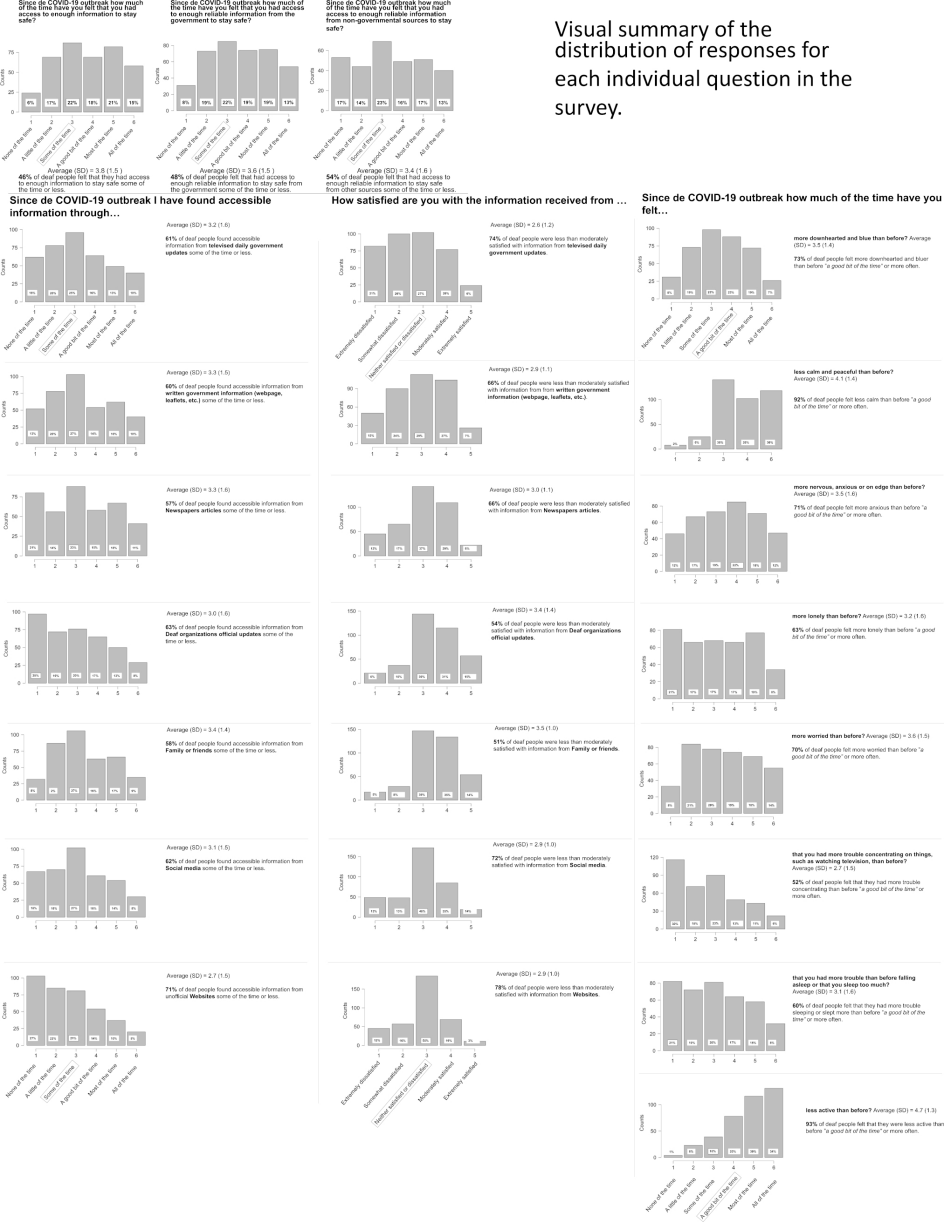
Visual summary of the percentages and distribution of responses for each survey question.

In this section, we first describe the modalities in which deaf/HoH people accessed information from different sources during the COVID-19 pandemic. The responses to the question “for each of the following sources of information about COVID-19 in which way have you found most of the information?” revealed that most deaf/HoH people relied on subtitles for information from the government and from the news, with text and SL right after. Deaf/HoH people mainly used text to access information on social media and websites, and sign language was the preferred modality to access information from deaf organisations. (see Fig 3 for the distribution of responses). Some participants selected ‘none of these modalities’ for each source, reflecting that they did not access information from that source. Congruent with this behaviour, the highest value for ‘none of these modalities’ was for deaf organisations, possibly because this is not the default option for late-onset HoH non-signers or in general for deaf/HoH people without a deaf identity. Having shown in these descriptive analyses that there is a wide variety of modalities of access to these sources, and that they all were accessed in some way, we now describe the response trends and report the analyses results for the rest of our dependent variables. In Table 2 we report the percentage frequency in each type of response for all questions (except SF-12) and in Fig 4 we visually show the distribution of responses for each survey question. Then group the question in three major sections, accessibility of information to stay safe, sources of accessible information, and physical and mental health and proceed to report trends and results within each of them.

**Table 2:** Percentage frequency in each response option for the survey questions.

Percentage frequency for each response option	None of the time	A little of the time	Some of the time	A good bit of the time	Most of the time	All of the time	Missing
**How much of the time have you felt that you had access to enough information to stay safe?**	6.1	17.4	21.97	17.4	20.7	14.6	1.8
**How much of the time have you felt that you had access to enough reliable information from the government to stay safe?**	7.8	18.4	21.5	18.7	18.9	13.6	1
**How much of the time have you felt that you had access to enough reliable information from non-governmental sources to stay safe?**	13.4	11.1	17.4	12.4	12.9	10.1	22.7
**I have found accessible information through…**	None of the time	A little of the time	Some of the time	A good bit of the time	Most of the time	All of the time	Missing
TV government updates	15.7	19.7	24.2	16.2	12.4	10.1	1.8
Written government information	13.1	19.7	26	13.6	15.7	10.1	1.8
Newspapers	20.2	14.1	22.2	14.6	16.9	10.4	1.5
Deaf organisations	24.5	18.2	19.2	16.4	12.6	7.3	1.8
Family	8.1	21.97	26.8	15.9	16.7	8.8	1.8
Social media	16.9	17.7	25.8	15.4	13.6	7.6	3
Websites	26	21.5	20.5	13.6	9.3	5.1	4
**How satisfied are you with the information received from…**	Extremely dissatisfied	Somewhat dissatisfied	Neither satisfied or dissatisfied	Moderately satisfied	Extremely satisfied		Missing
TV government updates	20.7	25.3	25.8	19.4	6.1		2.8
Written government information	12.6	22.7	28.5	26.3	6.6		3.3
Newspapers	11.4	16.4	35.9	27.5	5.6		3.3
Deaf organisations	5.3	9.3	36.4	29	14.4		5.6
Family	4.3	7.3	37.1	33.8	13.6		3.8
Social media	12.4	12.1	43.4	21.5	4.8		5.8
Websites	11.4	14.4	46.7	17.4	2.8		7.3
**Since the COVID-19 outbreak how much of the time have you felt …. than before?**	None of the time	A little of the time	Some of the time	A good bit of the time	Most of the time	All of the time	Missing
more sad	7.8	18.4	24.7	22.2	18.2	6.6	2
less calm	2	6.3	35.4	25.8	29.8	0.8	2
more nervous, anxious or on edge	11.6	16.9	18.4	21.5	17.9	11.9	1.8
more lonely	20.5	16.7	17.2	16.7	19.4	8.6	1
more worried	8.3	21.2	19.7	18.7	17.4	13.9	0.8
that you had more trouble concentrating on things	29.3	17.9	22.7	12.4	10.9	5.6	1.3
that you had more trouble falling asleep	20.7	18.2	20.5	16.2	14.6	8.1	1.8
less active	1	5.8	9.8	19.7	29.3	33.1	1.3

All statistical analyses were conducted in R (version 2024.04.1) employing the following packages: tidyverse and janitor for data cleaning; psych and GPArotation for exploratory factor analysis (EFA) using polychoric correlations and oblimin rotation; lavaan for confirmatory factor analysis (CFA) and structural equation modelling (SEM) with the WLSMV estimator; and semTools for reliability indices and factor score extraction.

Answers to questions in each of the sections below were submitted first to an Exploratory Factor Analysis (EFA) to examine the dimensionality of the corresponding items. We used EFA to reduce the number of dependent variables and improve measurement reliability as it allows us to identify latent constructs within blocks of ordinal survey items. The EFA was conducted using polychoric correlations, minimum residual extraction, and oblimin rotation, which is theoretically expected in social and health constructs. The number of factors was determined by parallel analysis combined with interpretability criteria. Items were retained if they showed primary loadings ≥ .40 and minimal cross-loadings (<.30). Internal consistency for each factor was assessed using McDonald’s omega (ω). Missing data were handled using pairwise deletion.

Following the EFA, we tested the resulting structures with CFA and fitted the EFA loading using WLSMV estimator in lavaan, which is recommended for ordinal indicators and missing data. Model fit was evaluated using CFI, TLI, RMSEA, and SRMR. Factor scores were estimated using the Empirical Bayes method and used as dependent variables in subsequent regression models. This two-step EFA–CFA approach ensures construct validity and reduces multiplicity while preserving theoretical meaning.

Finally, Structural Equation Modelling (SEM) was conducted using the lavaan and semTools packages in R for reliability and probing, with the WLSMV estimator to accommodate ordinal indicators and pairwise missing data. SEM simultaneously estimated both the measurement model (confirming latent constructs via CFA) and the structural model testing the effects of contrast-coded predictors (Residence (−1 = Spain, 1 = UK), Level of Deafness (−1 = HoH and 1 = deaf), Onset of Deafness (−1 = late, 1 = early), Knowledge of Sign Language (SL; −1 = non-signer, 1 = signer), and SA Reading Skill (−1 = less-skilled, 1 = skilled), and their interactions on latent outcomes providing standardized path coefficients, fit indices (χ², CFI, TLI, RMSEA, SRMR), and composite reliability (ρC). This approach was chosen because SEM allows for the modelling of latent variables while accounting for measurement error, supports complex interaction testing, and is robust to non-normal ordinal data. To examine the effects of interactions among contrast-coded predictors on latent outcomes, all possible two-way and three-way interaction terms were computed as products of the five original contrast-coded variables (−1/ + 1 coding). Interaction terms were incorporated into the structural equation models (SEM) as predictors of latent variables. Significant interactions were identified using standardized regression coefficients and z-tests and were further probed using simple effects analysis. This involved computing the effect of one variable at different levels of the other(s), using label-aware linear combinations of estimated parameters. Multiple testing corrections were applied using Benjamini-Hochberg (FDR) and Holm procedures, both within each latent outcome (or reliability enhanced composite) and across all planned tests (the data and R scripts, can be found in https://osf.io/gzdt9/?viewonly=abc0e87dacea42978f21973370eaf0c8). Note that due to the large number of interaction terms included and the sample including uneven subgroup cell sizes, the interaction effects, especially three‑way interactions, will be interpreted as exploratory and hypothesis‑generating rather than as confirmatory evidence. We also used SEM to analyse separately the items that did not load into any factor. For each sub-section, we first describe the trends for the responses to the individual questions (see Fig 4 for the visual comprehensive summary of responses). We then report the analyses.

### Accessibility of information necessary to stay safe

#### Description of trends.

Results showed that during the pandemic over 40% of deaf/HoH people felt that they had enough information to stay safe only some of the time or less.

#### Factor analysis.

EFA’s parallel analysis suggested a one-factor solution, which explained 62% of the variance. All three items loaded strongly on this factor (loadings = .81, .94, and .56), supporting a unidimensional structure. CFA tested this one-factor structure. The model was just identified (df = 0), resulting in perfect fit indices by definition (χ²(0) = 0; CFI = 1.00; TLI = 1.00; SRMR = 0.00; RMSEA = 0.00). However, note that in just identified models model fit indices are uninformative, interpretation therefore relies primarily on the strength of the loadings and the internal consistency and reliability estimates, and the resulting factor is best viewed as a reliability‑enhanced composite of the three items rather than a strongly validated construct. Standardized loadings were strong: general access to information to stay safe (.81), access to official information (.94), and access to non-official information (.56). Factor reliability was good (ω = .79; ordinal α = .81), and the average variance extracted (AVE = .62) exceeded the recommended .50 threshold, supporting convergent validity. Overall, the CFA corroborated the EFA’s one-factor solution. This factor was the dependent variable in subsequent SEM analyses with the coded predictors (Residence (−1 = Spain, 1 = UK), Level of Deafness (−1 = HoH and 1 = deaf), Onset of Deafness (−1 = late, 1 = early), knowledge of sign language (−1 = non-signer, 1 = signer), and SA Reading Skill (−1 = less-skilled, 1 = skilled).

#### The role of predictors.

The SEM analyses showed a main effect of Residence, Knowledge of SL, and SA Reading Skill. UK Residence (vs. Spain) predicted higher accessibility (b = 0.43, SE = 0.15, z = 2.94, p = .003, β = .44); sign language users (vs. non-signers) reported lower accessibility (b = −0.40, SE = 0.16, z = −2.58, p = .010, β = −.40); and skilled readers (vs. less-skilled) reported higher accessibility (b = 0.35, SE = 0.13, z = 2.72, p = .006, β = .35). There were significant interactions between sign language knowledge and Reading Skill (b = 0.35, p = .015), Residence and Reading Skill (b = −0.29, p = .032), and Level of Deafness, Onset of Deafness, and Reading Skill (b = 0.20, p = .044). The results of the SEM analyses are summarised in Table 3.

**Table 3:** Summary of results on access to information to stay safe.

hs	rhs	est	se	z	p	std.all
**Accessibility of information necessary to stay safe**	**Knowledge of SL**	**−0.402**	**0.156**	**−2.58**	**0.01**	−0.396
	Knowledge of SL * SA Reading Skill	**0.349**	**0.144**	**2.428**	**0.015**	0.383
	Level of Deafness	−0.037	0.134	−0.276	0.783	−0.036
	Level of Deafness * Knowledge of SL	−0.024	0.148	−0.161	0.872	−0.023
	Level of Deafness * Knowledge of SL * SA Reading Skill	−0.186	0.146	−1.277	0.202	−0.203
	Level of Deafness * Onset of Deafness	0.002	0.132	0.015	0.988	0.002
	Level of Deafness * Onset of Deafness * Knowledge of SL	−0.012	0.074	−0.169	0.866	−0.013
	**Level of Deafness** * **Onset of Deafness** * **SA Reading Skill**	**0.204**	**0.101**	**2.011**	**0.044**	0.222
	Level of Deafness * SA Reading Skill	−0.005	0.114	−0.045	0.964	−0.006
	Onset of Deafness	0.163	0.126	1.296	0.195	0.166
	Onset of Deafness * Knowledge of SL	0.254	0.162	1.566	0.117	0.226
	Onset of Deafness * Knowledge of SL * SA Reading Skill	−0.178	0.141	−1.262	0.207	−0.194
	Onset of Deafness * SA Reading Skill	−0.209	0.119	−1.764	0.078	−0.231
	**Residence**	**0.433**	**0.148**	**2.938**	**0.003**	0.443
	Residence * Knowledge of SL	0.124	0.167	0.743	0.458	0.134
	Residence * Knowledge of SL * SA Reading Skill	−0.084	0.131	−0.638	0.523	−0.092
	Residence * Level of Deafness	−0.158	0.111	−1.422	0.155	−0.173
	Residence * Level of Deafness * Knowledge of SL	0.158	0.094	1.676	0.094	0.174
	Residence * Level of Deafness * Onset of Deafness	−0.131	0.087	−1.503	0.133	−0.144
	Residence * Level of Deafness * SA Reading Skill	0.193	0.116	1.659	0.097	0.212
	Residence * Onset of Deafness	−0.328	0.195	−1.682	0.093	−0.362
	Residence * Onset of Deafness * Knowledge of SL	−0.149	0.097	−1.542	0.123	−0.162
	Residence * Onset of Deafness * SA Reading Skill	0.303	0.191	1.588	0.112	0.334
	**Residence** * **SA Reading Skill**	**−0.288**	**0.135**	**−2.141**	**0.032**	−0.298
	**SA Reading Skill**	**0.354**	**0.13**	**2.722**	**0.006**	0.352

The interaction between knowledge of sign language and SA Reading Skill showed that among less-skilled readers, sign language users reported significantly lower accessibility compared to non-signers (b = −1.50, SE = 0.55, z = −2.74, p = .006, q = .012). In contrast, among skilled readers, the effect of sign language knowledge was non-significant (b = −0.11, p = .661). Similarly, among signers skilled readers had access to more information than less-skilled readers (b = 1.40, SE = 0.44, z = 3.20, p = .001, q = .006), but there were no significant differences among non-signers (b = 0.01, p = .977).

The interaction between Residence and Reading Skill showed that among less-skilled readers, UK participants reported significantly higher accessibility than those in Spain (b = 1.44, SE = 0.53, z = 2.70, p = .007, q = .014). However, among skilled readers, the effect of country was not significant (b = 0.29, p = .112). Conversely, Reading Skill was significantly associated with higher accessibility in Spain (b = 1.28, SE = 0.42, z = 3.09, p = .002, q = .008), but not in the UK (b = 0.13, p = .692).

The three-way interaction between Level of Deafness, Onset of Deafness, and Reading Skill revealed a significant effect only for one subgroup. Specifically, skilled readers who were HoH with late-onset deafness reported significantly higher accessibility than less-skilled HoH late onset readers (b = 1.54, SE = 0.53, z = 2.90, p = .004, q = .045). All other combinations of deafness level, onset, and Reading Skill did not reach statistical significance (all q-values > .13).

### Sources of accessible information

#### Description of trends.

Results showed that around 60% of deaf/HoH people found accessible information only some of the time or less from the government, this included the TV updates (61%) as well as other written information (60%). Similar percentages of people found accessible information only *some or the time or less* in newspapers (57%), deaf organisations (63%), social media (62%) and family or friends (58%). The proportion was larger for websites, where 71% of deaf/HoH people found accessible information only some of the time or less.

#### Factor analysis.

The EFA examined the dimensionality of the items (*Televised daily government updates / Written government information (webpage, leaflets, etc) / Newspapers articles / Deaf organisations official updates / Family or friends / Unofficial: Social media / Unofficial: Websites).* Parallel analysis suggested a three-factor solution, which explained 58% of the variance. Items related to community sources (social media, websites, family) loaded on Factor 1, official sources (written government information, newspapers, *televised daily government updates*) loaded on Factor 2, and deaf organisations loaded on Factor 3. The CFA conducted to evaluate the initial three-factor structure derived from the revised exploratory factor analysis (EFA) indicated poor model fit due to one factor comprising only items related to deaf organisations. A revised CFA was then conducted to evaluate a two-factor solution. This model demonstrated acceptable fit: χ² [8] = 20.62, p = .008, CFI = .99, TLI = .99, RMSEA = .06, 90% CI [.03, .10], SRMR = .05. Standardized factor loadings ranged from .52 to .87. Composite reliability was adequate for Factor 1 (ordinal α = .77; ω = .77; AVE = .57), but lower for Factor 2 (ordinal α = .66; ω = .64; AVE = .41), suggesting weaker internal consistency for official sources. The latent factor correlation was modest (r = .31), indicating that digital and official sources are related but distinct constructs. The CFA partially confirmed the EFA-informed structure: items related to digital sources (websites, social media, family) and official sources (written government information, newspapers, television) clustered as expected. These two factors and deaf organisations were the dependent variables of further analyses with the coded predictors (Residence (−1 = Spain, 1 = UK), Level of Deafness (−1 = HoH and 1 = deaf), Onset of Deafness (−1 = late, 1 = early), knowledge of sign language (−1 = non-signer, 1 = signer), and SA Reading Skill (−1 = less-skilled, 1 = skilled).

#### The role of predictors.

**Official sources**: SA Reading Skill was significantly associated with higher perceived accessibility of official sources (b = 0.20, SE = 0.10, z = 2.70, p = .006, β = .40). Additionally, the interaction between Residence, Level of Deafness, and knowledge of sign language was significant (b = −0.10, SE = 0.05, z = −2.00, p = .042, β = −.20). Analysis of the interaction indicated that among deaf participants in the UK, signers reported lower accessibility than non-signers (b = −0.44, SE = 0.20, z = −2.21, p = .027, q = .327). All other contrasts were not significant (p > .05) (Table 4).

**Table 4:** Summary of results on sources of accessible information: official sources factor.

hs	rhs	est	se	z	p	std.all
**Official sources (Written government info, Newspapers, TV government updates)**	Knowledge of SL	−0.124	0.135	−0.917	0.359	−0.197
	Knowledge of SL * SA Reading Skill	0.107	0.136	0.784	0.433	0.19
	Level of Deafness	0.083	0.074	1.131	0.258	0.132
	Level of Deafness * Knowledge of SL	−0.125	0.117	−1.066	0.287	−0.2
	Level of Deafness * Knowledge of SL * SA Reading Skill	0.209	0.118	1.762	0.078	0.369
	Level of Deafness * Onset of Deafness	0.084	0.102	0.828	0.408	0.134
	Level of Deafness * Onset of Deafness * Knowledge of SL	−0.021	0.051	−0.408	0.683	−0.034
	Level of Deafness * Onset of Deafness * SA Reading Skill	−0.1	0.098	−1.024	0.306	−0.176
	Level of Deafness * SA Reading Skill	−0.085	0.067	−1.281	0.2	−0.151
	Onset of Deafness	0.013	0.129	0.098	0.922	0.021
	Onset of Deafness * Knowledge of SL	0.128	0.098	1.303	0.193	0.184
	Onset of Deafness * Knowledge of SL * SA Reading Skill	−0.136	0.086	−1.586	0.113	−0.24
	Onset of Deafness * SA Reading Skill	0.007	0.125	0.058	0.954	0.013
	Residence	−0.077	0.082	−0.935	0.35	−0.127
	Residence* Knowledge of SL	0.145	0.144	1.006	0.314	0.255
	Residence* Knowledge of SL * SA Reading Skill	−0.189	0.144	−1.306	0.191	−0.335
	Residence * Level of Deafness	0.021	0.064	0.335	0.737	0.038
	Residence * Level of Deafness * Knowledge of SL	**−0.116**	**0.057**	**−2.034**	**0.042**	−0.207
	Residence * Level of Deafness * Onset of Deafness	0.029	0.053	0.549	0.583	0.052
	Residence * Level of Deafness * SA Reading Skill	0.024	0.071	0.34	0.734	0.043
	Residence * Onset of Deafness	−0.193	0.15	−1.283	0.199	−0.345
	Residence * Onset of Deafness * Knowledge of SL	−0.057	0.057	−0.995	0.32	−0.1
	Residence * Onset of Deafness * SA Reading Skill	0.18	0.148	1.222	0.222	0.322
	Residence * SA Reading Skill	0.035	0.079	0.446	0.656	0.059
	**SA Reading Skill**	**0.25**	**0.091**	**2.737**	**0.006**	0.403

**Community sources**: Residence (UK vs. Spain) was significantly associated with lower scores (b = −0.30, SE = 0.10, z = −3.40, p = .001, β = −.50), indicating reduced perceived accessibility in the UK. No other predictors or interactions reached statistical significance (all p-values > .05) (Table 5).

**Table 5:** Summary of results on sources of accessible information for the community sources factor.

hs	rhs	est	se	z	p	std.all
**Community sources (social media, websites, family)**	Knowledge of SL	0.018	0.089	0.208	0.835	0.028
	Knowledge of SL * SA Reading Skill	−0.08	0.097	−0.82	0.412	−0.135
	Level of Deafness	−0.044	0.08	−0.553	0.58	−0.067
	Level of Deafness * Knowledge of SL	0.083	0.104	0.796	0.426	0.126
	Level of Deafness * Knowledge of SL * SA Reading Skill	−0.01	0.115	−0.091	0.927	−0.018
	Level of Deafness * Onset of Deafness	0.059	0.11	0.537	0.592	0.089
	Level of Deafness * Onset of Deafness * Knowledge of SL	−0.037	0.053	−0.704	0.482	−0.058
	Level of Deafness * Onset of Deafness * SA Reading Skill	−0.073	0.103	−0.709	0.479	−0.123
	Level of Deafness * SA Reading Skill	0.056	0.081	0.694	0.488	0.094
	Onset of Deafness	0.013	0.093	0.143	0.886	0.021
	Onset of Deafness * Knowledge of SL	−0.043	0.093	−0.461	0.645	−0.059
	Onset of Deafness * Knowledge of SL * SA Reading Skill	0.034	0.081	0.414	0.679	0.057
	Onset of Deafness * SA Reading Skill	−0.009	0.088	−0.107	0.915	−0.016
	**Residence**	**−0.287**	**0.085**	**−3.384**	**0.001**	−0.452
	Residence* Knowledge of SL	0.025	0.109	0.231	0.817	0.042
	Residence* Knowledge of SL * SA Reading Skill	−0.007	0.106	−0.07	0.944	−0.013
	Residence * Level of Deafness	0.122	0.064	1.917	0.055	0.207
	Residence * Level of Deafness * Knowledge of SL	−0.064	0.073	−0.885	0.376	−0.109
	Residence * Level of Deafness * Onset of Deafness	0.028	0.071	0.392	0.695	0.047
	Residence * Level of Deafness * SA Reading Skill	−0.01	0.082	−0.116	0.907	−0.016
	Residence * Onset of Deafness	0.018	0.124	0.142	0.887	0.03
	Residence * Onset of Deafness * Knowledge of SL	0.039	0.06	0.656	0.512	0.066
	Residence * Onset of Deafness * SA Reading Skill	−0.001	0.123	−0.01	0.992	−0.002
	Residence * SA Reading Skill	0.064	0.079	0.814	0.416	0.102
	SA Reading Skill	0.067	0.093	0.725	0.468	0.104

**Deaf organisations:** There was a three‑way interaction between Residence, knowledge of sign language, and Reading Skill (b = 0.29, SE = 0.11, z = 2.58, p = .010, β = .25). Analysis of the interaction showed that for non-signers who were skilled readers accessibility was higher in the UK than in Spain (*b* = −0.75, *SE* = 0.29, *z* = −2.56, *p* = .010, *q* = .063); for non-signers who were less-skilled readers accessibility was higher in Spain than in the UK (*b* = 1.23, *SE* = 0.62, *z* = 1.97, *p* = .048, *q* = .140). Furthermore, in the UK among skilled readers, signers reported higher accessibility than non-signers (*b* = 0.70, *SE* = 0.25, *z* = 2.86, *p* = .004, *q* = .051); and in the UK among non-signers, skilled readers reported lower accessibility than less-skilled readers (*b* = −1.26, *SE* = 0.57, *z* = −2.20, *p* = .028, *q* = .111). All other contrasts were not significant (*ps* > .05).

There also was a three‑way interaction between Onset of Deafness, knowledge of sign language, and Reading Skill (b = 0.34, SE = 0.11, z = 3.22, p = .001, β = .29). Analysis of the interaction showed that for non-signers who were less-skilled readers accessibility was higher for those with late onset than early Onset of Deafness (*b* = 1.22, *SE* = 0.53, *z* = 2.29, *p* = .022, *q* = .132); among less-skilled readers with late onset deafness, signers reported higher accessibility than non-signers (*b* = 2.45, *SE* = 0.97, *z* = 2.54, *p* = .011, *q* = .132). All other contrasts were not significant (*ps* > .05).

All other main effects and interactions were not significant (all p-values > .05) (Table 6).

**Table 6:** Summary of results on sources of accessible information from deaf organisations.

hs	rhs	est	se	z	p	std.all
**Deaf organisations**	Knowledge of SL	0.444	0.26	1.706	0.088	0.343
	Knowledge of SL * SA Reading Skill	−0.058	0.163	−0.355	0.723	−0.05
	Level of Deafness	0.411	0.234	1.756	0.079	0.317
	Level of Deafness * Knowledge of SL	0.119	0.257	0.463	0.643	0.092
	Level of Deafness * Knowledge of SL * SA Reading Skill	−0.178	0.163	−1.093	0.274	−0.152
	Level of Deafness * Onset of Deafness	−0.176	0.123	−1.427	0.153	−0.135
	Level of Deafness * Onset of Deafness * Knowledge of SL	−0.071	0.116	−0.609	0.543	−0.056
	Level of Deafness * Onset of Deafness * SA Reading Skill	0.206	0.118	1.754	0.079	0.176
	Level of Deafness * SA Reading Skill	−0.124	0.122	−1.021	0.307	−0.106
	Onset of Deafness	−0.073	0.183	−0.401	0.688	−0.058
	Onset of Deafness * Knowledge of SL	**−0.383**	**0.193**	**−1.99**	**0.047**	**−0.267**
	Onset of Deafness * Knowledge of SL * SA Reading Skill	**0.341**	**0.106**	**3.223**	**0.001**	**0.291**
	Onset of Deafness * SA Reading Skill	0.039	0.123	0.318	0.751	0.034
	Residence	−0.205	0.252	−0.813	0.416	−0.164
	Residence* Knowledge of SL	−0.325	0.233	−1.397	0.162	−0.277
	Residence* Knowledge of SL * SA Reading Skill	**0.292**	**0.113**	**2.583**	**0.01**	**0.251**
	Residence * Level of Deafness	−0.19	0.255	−0.748	0.455	−0.164
	Residence * Level of Deafness * Knowledge of SL	−0.016	0.22	−0.074	0.941	−0.014
	Residence * Level of Deafness * Onset of Deafness	0.212	0.131	1.625	0.104	0.183
	Residence * Level of Deafness * SA Reading Skill	0.051	0.168	0.303	0.762	0.044
	Residence * Onset of Deafness	0.183	0.207	0.884	0.377	0.158
	Residence * Onset of Deafness * Knowledge of SL	0.174	0.158	1.098	0.272	0.148
	Residence * Onset of Deafness * SA Reading Skill	−0.187	0.145	−1.286	0.198	−0.162
	Residence * SA Reading Skill	−0.202	0.144	−1.41	0.159	−0.164
	SA Reading Skill	−0.196	0.136	−1.443	0.149	−0.153

### Satisfaction levels with the different sources of information

#### Description of trends.

Only around 30% of deaf/HoH people were moderately or extremely satisfied with the information received from the government TV updates (26%), social media (28%) and websites (22%). Over 30% of deaf/HoH people were moderately or extremely satisfied with written information distributed by the government (34%) and in newspapers (35%). More deaf/HoH people were moderately or extremely satisfied with the information received from deaf organisations (46%) and their own family and friends (49%).

#### Factor analysis.

EFA examined the dimensionality of the items (Televised daily government updates / Written government information (webpage, leaflets, etc) / Newspapers articles / Deaf organisations official updates / Family or friends / Unofficial: Social media / Unofficial: Websites). Parallel analysis suggested a two-factor solution, which explained 50% of the variance. Items related to official sources (television, written government information, newspapers) loaded on Factor 1, while items related to digital and community sources (social media, websites, family) loaded on Factor 2. The item on deaf organisations did not load strongly on either factor. Confirmatory factor analysis (CFA) tested the two-factor structure. Model fit was mixed—χ² [8] = 52.33, p < .001; CFI = .982; TLI = .967; SRMR = .074; RMSEA = .120, 90% CI [.090, .151]. Despite the elevated RMSEA (which can be inflated in low–degrees-of-freedom models), incremental fit indices indicated excellent fit. Standardized loadings were strong for official sources (written government info = .92; TV = .78; newspapers = .59) and community sources (social media = .84; websites = .79; family = .47). Factor reliability was acceptable (ω = .79 and .71; ordinal α = .79 and .71), with AVE ≥ .50 for both constructs (F1 = .60; F2 = .51), supporting convergent validity. The factors were moderately correlated (φ = .35), and Fornell–Larcker criteria were met (AVE > φ²), supporting discriminant validity. Overall, the CFA corroborated the EFA’s two-factor solution. The item on deaf organisations will be further analysed separately.

#### The role of predictors.

**Official sources**: Skilled readers reported greater satisfaction (b = 0.37, SE = 0.17, z = 2.23, p = .026, β = .39), while signers reported lower satisfaction (b = −0.34, SE = 0.16, z = −2.18, p = .029, β = −.35). Additionally, UK residents reported higher satisfaction compared to Spanish residents (b = 0.28, SE = 0.13, z = 2.10, p = .036, β = .30). There was a significant interaction of Residence and Onset of Deafness (b = −0.38, SE = 0.17, z = −2.21, p = .027, β = −.44), and a three-way interaction between Residence, Onset of Deafness, and Reading Skill (b = 0.36, SE = 0.17, z = 2.13, p = .033, β = .42).

Analysis of the interaction revealed that among people with late-onset deafness and lower Reading Skill, those from Spain reported higher satisfaction than those from the UK (b = 2.50, SE = 0.95, z = 2.61, p = .009, q = .064). Furthermore, among Spanish participants with late-onset deafness, those with higher Reading Skill reported greater satisfaction than those with lower Reading Skill (b = 2.38, SE = 0.93, z = 2.56, p = .011, q = .064). No other interaction effects reached statistical significance (all q-values > .08) (Table 7).

**Table 7:** Summary of results on satisfaction for official sources factor.

hs	rhs	est	se	z	p	std.all
**Official sources (Written government info, Newspapers, TV government updates)**	**Knowledge of SL**	**−0.341**	**0.157**	**−2.179**	**0.029**	−0.354
	Knowledge of SL * SA Reading Skill	0.179	0.171	1.045	0.296	0.206
	Level of Deafness	0.029	0.118	0.242	0.809	0.029
	Level of Deafness * Knowledge of SL	0.081	0.146	0.555	0.579	0.084
	Level of Deafness * Knowledge of SL * SA Reading Skill	−0.068	0.16	−0.424	0.671	−0.078
	Level of Deafness * Onset of Deafness	0.068	0.134	0.508	0.611	0.07
	Level of Deafness * Onset of Deafness * Knowledge of SL	−0.03	0.076	−0.396	0.692	−0.032
	Level of Deafness * Onset of Deafness * SA Reading Skill	−0.004	0.12	−0.034	0.973	−0.005
	Level of Deafness * SA Reading Skill	0.049	0.124	0.395	0.693	0.056
	Onset of Deafness	0.074	0.137	0.539	0.59	0.079
	Onset of Deafness * Knowledge of SL	0.299	0.16	1.862	0.063	0.279
	Onset of Deafness * Knowledge of SL * SA Reading Skill	−0.202	0.121	−1.664	0.096	−0.231
	Onset of Deafness * SA Reading Skill	−0.226	0.152	−1.492	0.136	−0.263
	**Residence**	**0.282**	**0.134**	**2.102**	**0.036**	0.304
	Residence* Knowledge of SL	0.286	0.174	1.644	0.1	0.327
	Residence* Knowledge of SL * SA Reading Skill	−0.189	0.146	−1.288	0.198	−0.218
	Residence * Level of Deafness	0.008	0.119	0.066	0.947	0.009
	Residence * Level of Deafness * Knowledge of SL	−0.203	0.112	−1.812	0.07	−0.235
	Residence * Level of Deafness * Onset of Deafness	0.013	0.105	0.121	0.903	0.015
	Residence * Level of Deafness * SA Reading Skill	0.144	0.124	1.167	0.243	0.167
	Residence * Onset of Deafness	**−0.376**	**0.17**	**−2.211**	**0.027**	−0.437
	Residence * Onset of Deafness * Knowledge of SL	−0.15	0.09	−1.67	0.095	−0.172
	Residence * Onset of Deafness * SA Reading Skill	**0.363**	**0.17**	**2.134**	**0.033**	0.421
	Residence * SA Reading Skill	−0.226	0.139	−1.632	0.103	−0.246
	**SA Reading Skill**	**0.374**	**0.168**	**2.229**	**0.026**	0.392

**Community sources**: A significant three-way interaction was found between Residence, Level of Deafness, and SA Reading Skill, b = 0.13, SE = 0.06, z = 2.23, p = .026, β = .27. No other predictors or interactions reached statistical significance (all p-values > .05). Analysis of the interaction showed no significant effects (all ps > .05) (Table 8).

**Table 8:** Summary of results on satisfaction for community sources factor.

hs	rhs	est	se	z	p	std.all
**Community sources (social media, websites, family)**	Knowledge of SL	−0.011	0.102	−0.108	0.914	−0.02
	Knowledge of SL * SA Reading Skill	−0.087	0.103	−0.844	0.399	−0.175
	Level of Deafness	−0.009	0.07	−0.131	0.896	−0.017
	Level of Deafness * Knowledge of SL	0.023	0.115	0.204	0.838	0.043
	Level of Deafness * Knowledge of SL * SA Reading Skill	−0.1	0.116	−0.867	0.386	−0.201
	Level of Deafness * Onset of Deafness	0.027	0.11	0.241	0.81	0.048
	Level of Deafness * Onset of Deafness * Knowledge of SL	0.009	0.051	0.175	0.861	0.017
	Level of Deafness * Onset of Deafness * SA Reading Skill	0.054	0.107	0.503	0.615	0.107
	Level of Deafness * SA Reading Skill	0.016	0.061	0.259	0.796	0.032
	Onset of Deafness	0.023	0.094	0.247	0.805	0.043
	Onset of Deafness * Knowledge of SL	−0.039	0.104	−0.371	0.711	−0.063
	Onset of Deafness * Knowledge of SL * SA Reading Skill	0.09	0.097	0.933	0.351	0.18
	Onset of Deafness * SA Reading Skill	0.014	0.093	0.15	0.881	0.028
	Residence	0.005	0.072	0.076	0.94	0.01
	Residence* Knowledge of SL	−0.048	0.115	−0.419	0.675	−0.096
	Residence* Knowledge of SL * SA Reading Skill	0.164	0.114	1.441	0.15	0.329
	Residence * Level of Deafness	−0.081	0.055	−1.476	0.14	−0.163
	Residence * Level of Deafness * Knowledge of SL	−0.033	0.054	−0.607	0.544	−0.066
	Residence * Level of Deafness * Onset of Deafness	0.071	0.053	1.321	0.186	0.142
	Residence * Level of Deafness * SA Reading Skill	**0.134**	**0.06**	**2.23**	**0.026**	0.271
	Residence * Onset of Deafness	0.056	0.126	0.446	0.655	0.114
	Residence * Onset of Deafness * Knowledge of SL	−0.042	0.052	−0.818	0.414	−0.084
	Residence * Onset of Deafness * SA Reading Skill	−0.132	0.123	−1.069	0.285	−0.267
	Residence * SA Reading Skill	−0.033	0.065	−0.506	0.613	−0.062
	SA Reading Skill	0.012	0.099	0.117	0.907	0.021

**Deaf organisations:** There was a three‑way interaction between Onset of Deafness, knowledge of sign language, and Reading Skill (b = 0.31, SE = 0.16, z = 1.98, p = .048, β = .28). Analysis of the interaction showed that among signers with early Onset of Deafness skilled readers reported higher satisfaction than less-skilled readers (*b* = 0.77, *SE* = 0.33, *z* = 2.33, *p* = .020, *q* = .238). All other contrasts were not significant (*ps* > .05) (Table 9).

**Table 9:** Summary of results on satisfaction for deaf organisations.

hs	rhs	est	se	z	p	std.all
**Deaf organisations**	Knowledge of SL	0.125	0.227	0.551	0.582	0.103
	Knowledge of SL * SA Reading Skill	0.137	0.199	0.692	0.489	0.126
	Level of Deafness	0.226	0.163	1.388	0.165	0.186
	Level of Deafness * Knowledge of SL	0.34	0.195	1.742	0.082	0.282
	Level of Deafness * Knowledge of SL * SA Reading Skill	−0.24	0.199	−1.211	0.226	−0.22
	Level of Deafness * Onset of Deafness	0.009	0.187	0.046	0.964	0.007
	Level of Deafness * Onset of Deafness * Knowledge of SL	−0.169	0.144	−1.177	0.239	−0.143
	Level of Deafness * Onset of Deafness * SA Reading Skill	0.015	0.176	0.086	0.932	0.014
	Level of Deafness * SA Reading Skill	−0.111	0.138	−0.805	0.421	−0.102
	Onset of Deafness	−0.066	0.211	−0.315	0.753	−0.057
	Onset of Deafness * Knowledge of SL	−0.328	0.186	−1.764	0.078	−0.244
	Onset of Deafness * Knowledge of SL * SA Reading Skill	**0.31**	**0.156**	**1.981**	**0.048**	**0.283**
	Onset of Deafness * SA Reading Skill	0.022	0.191	0.117	0.907	0.021
	Residence	0.02	0.174	0.112	0.911	0.017
	Residence* Knowledge of SL	−0.112	0.22	−0.508	0.612	−0.101
	Residence* Knowledge of SL * SA Reading Skill	0.041	0.2	0.205	0.838	0.038
	Residence * Level of Deafness	−0.162	0.144	−1.126	0.26	−0.149
	Residence * Level of Deafness * Knowledge of SL	−0.128	0.141	−0.907	0.364	−0.117
	Residence * Level of Deafness * Onset of Deafness	0.161	0.136	1.184	0.237	0.148
	Residence * Level of Deafness * SA Reading Skill	0.188	0.137	1.376	0.169	0.173
	Residence * Onset of Deafness	0.173	0.22	0.788	0.431	0.16
	Residence * Onset of Deafness * Knowledge of SL	0.015	0.138	0.107	0.915	0.013
	Residence * Onset of Deafness * SA Reading Skill	−0.14	0.223	−0.63	0.529	−0.13
	Residence * SA Reading Skill	−0.156	0.15	−1.034	0.301	−0.135
	SA Reading Skill	−0.087	0.195	−0.445	0.656	−0.072

### Physical and mental health

The mean scores for the physical health were around 50, suggesting average physical health. The scores for the mental health component were slightly below 50, suggesting slightly below average mental health in comparison with the typical (non-deaf) population before the pandemic.

**Physical health:** There was an effect Onset of Deafness, early onset participants had better physical health than those with early late deafness (*b* = 7.02, *SE* = 2.36, *z* = 2.98, *p* = .003). There was also an effect of knowledge of sign language (*b* = −6.81, *SE* = 2.91, *z* = −2.34, *p* = .019), and a significant interaction between knowledge of sign language and Reading Skill (*b* = 5.32, *SE* = 2.55, *z* = 2.09, *p* = .037). Analysis of the interaction showed that among less-skilled readers, signers reported substantially lower physical health than non-signers (*b* = −24.26, *SE* = 10.17, *z* = −2.38, *p* = .017, *q* = .034), whereas among skilled readers, this difference was not significant (*p* = .458). Among signers, skilled readers reported higher physical health than less-skilled readers (*b* = 20.14, *SE* = 7.99, *z* = 2.52, *p* = .012, *q* = .034), while among non-signers, Reading Skill had no effect (*p* = .864) (Table 10).

**Table 10:** Summary of results on physical health.

hs	rhs	est	se	z	p	std.all
**Physical health**	**Knowledge of SL**	**−6.81**	**2.906**	**−2.343**	**0.019**	**−0.255**
	Knowledge of SL * SA Reading Skill	**5.32**	**2.552**	**2.085**	**0.037**	**0.222**
	Level of Deafness	2.748	2.121	1.295	0.195	0.102
	Level of Deafness * Knowledge of SL	−1.464	2.125	−0.689	0.491	−0.055
	Level of Deafness * Knowledge of SL * SA Reading Skill	−1.098	2.468	−0.445	0.656	−0.045
	Level of Deafness * Onset of Deafness	0.031	1.923	0.016	0.987	0.001
	Level of Deafness * Onset of Deafness * Knowledge of SL	−2.106	1.594	−1.322	0.186	−0.081
	Level of Deafness * Onset of Deafness * SA Reading Skill	0.673	1.899	0.354	0.723	0.028
	Level of Deafness * SA Reading Skill	0.431	1.963	0.219	0.826	0.018
	**Onset of Deafness**	**7.022**	**2.356**	**2.981**	**0.003**	**0.271**
	Onset of Deafness * Knowledge of SL	4.018	3.027	1.327	0.184	0.135
	Onset of Deafness * Knowledge of SL * SA Reading Skill	−3.187	2.569	−1.241	0.215	−0.132
	Onset of Deafness * SA Reading Skill	−3.19	2.191	−1.456	0.145	−0.133
	Residence	−0.775	2.644	−0.293	0.769	−0.03
	Residence* Knowledge of SL	1.698	2.318	0.732	0.464	0.07
	Residence* Knowledge of SL * SA Reading Skill	−0.937	1.77	−0.529	0.597	−0.039
	Residence * Level of Deafness	−0.89	2.15	−0.414	0.679	−0.037
	Residence * Level of Deafness * Knowledge of SL	2.517	1.936	1.3	0.194	0.105
	Residence * Level of Deafness * Onset of Deafness	1.572	1.819	0.864	0.388	0.065
	Residence * Level of Deafness * SA Reading Skill	1.981	1.988	0.996	0.319	0.083
	Residence * Onset of Deafness	−4.281	2.589	−1.654	0.098	−0.179
	Residence * Onset of Deafness * Knowledge of SL	−3.315	2.173	−1.526	0.127	−0.136
	Residence * Onset of Deafness * SA Reading Skill	0.497	2.706	0.184	0.854	0.021
	Residence * SA Reading Skill	0.293	2.616	0.112	0.911	0.011
	SA Reading Skill	4.75	2.653	1.79	0.073	0.18

**Mental health**: Signers had poorer mental health than non-signers (*b* = −5.95, *SE* = 3.03, *z* = −1.96, *p* = .049), and those with early onset deafness reported better mental health than those with late onset (*b* = 6.05, *SE* = 3.06, *z* = 1.98, *p* = .048). All other main and interaction effects were not significant (*ps* > .05) (Table 11).

**Table 11:** Summary of results on mental health.

hs	rhs	est	se	z	p	std.all
**Mental health**	**Knowledge of SL**	**−5.953**	**3.031**	**−1.964**	**0.049**	**−0.196**
	Knowledge of SL * SA Reading Skill	2.392	2.938	0.814	0.416	0.088
	Level of Deafness	2.464	2.248	1.096	0.273	0.081
	Level of Deafness * Knowledge of SL	−1.432	2.884	−0.497	0.619	−0.047
	Level of Deafness * Knowledge of SL * SA Reading Skill	2.663	2.981	0.893	0.372	0.097
	Level of Deafness * Onset of Deafness	3.204	3.009	1.065	0.287	0.105
	Level of Deafness * Onset of Deafness * Knowledge of SL	−0.356	1.800	−0.198	0.843	−0.012
	Level of Deafness * Onset of Deafness * SA Reading Skill	1.150	2.955	0.389	0.697	0.042
	Level of Deafness * SA Reading Skill	−0.790	2.165	−0.365	0.715	−0.029
	**Onset of Deafness**	**6.052**	**3.057**	**1.980**	**0.048**	**0.206**
	Onset of Deafness * Knowledge of SL	−0.157	3.377	−0.046	0.963	−0.005
	Onset of Deafness * Knowledge of SL * SA Reading Skill	−1.112	3.198	−0.348	0.728	−0.041
	Onset of Deafness * SA Reading Skill	−3.628	2.923	−1.241	0.215	−0.134
	Residence	−1.897	2.372	−0.800	0.424	−0.065
	Residence* Knowledge of SL	−0.102	3.552	−0.029	0.977	−0.004
	Residence* Knowledge of SL * SA Reading Skill	1.973	3.491	0.565	0.572	0.072
	Residence * Level of Deafness	3.465	1.957	1.770	0.077	0.127
	Residence * Level of Deafness * Knowledge of SL	0.971	1.828	0.531	0.595	0.036
	Residence * Level of Deafness * Onset of Deafness	−1.609	1.871	−0.860	0.390	−0.059
	Residence * Level of Deafness * SA Reading Skill	0.687	1.968	0.349	0.727	0.025
	Residence * Onset of Deafness	−4.672	3.943	−1.185	0.236	−0.173
	Residence * Onset of Deafness * Knowledge of SL	1.229	2.006	0.613	0.540	0.045
	Residence * Onset of Deafness * SA Reading Skill	2.328	4.101	0.568	0.570	0.086
	Residence * SA Reading Skill	−1.030	2.165	−0.476	0.634	−0.036
	SA Reading Skill	4.308	3.309	1.302	0.193	0.144

### Specific aspects of mental health

#### Description of trends.

Responses to the studied aspects of mental health showed that the majority of deaf/HoH people felt more downhearted and bluer (73%), less calm (92%) more anxious (71%), more lonely (63%), more worried (70%), having more trouble concentrating (52%), having a disrupted sleeping pattern (60%) and less active (93%) than before COVID-19 pandemic.

#### Factor analysis.

EFA examined the dimensionality of the items (“ a) “more downhearted and blue than before“, b) “less calm and peaceful than before, c) “more nervous, anxious or on edge than before, d) “more lonely than before, e) “more worried than before, f) “that you had more trouble concentrating on things, such as watching television, than before”, and g) “that you had more trouble than before falling asleep of that you sleep too much”.) Parallel analysis suggested a four-factor solution, which explained 77% of the variance. Items clustered into factors representing emotional distress (anxious, worried, less calm), cognitive strain (sleep disruption, concentration difficulties), sadness/loneliness, and physical inactivity. Given theoretical considerations and interpretability, this structure informed the specification of a confirmatory factor analysis (CFA) model. The CFA evaluated the four-factor structure derived from the revised EFA, the model had an unacceptable fit due to a factor including only less active. A revised three-factor CFA—excluding “less active”, fit the data excellently (χ² [11] = 5.74, p = .89, CFI = 1.00, TLI = 1.00, RMSEA = .00, 90% CI [.00, .03], SRMR = .02), with standardized loadings ranging from .53 to .95, acceptable reliability (ordinal α = .81–.83; ω = .78–.84), and substantial latent correlations (.75–.90). The item less active was analysed separately.

#### The role of predictors.

**Emotional distress** (anxious, worried, less calm): There were no significant predictors (Table 12).

**Table 12:** Summary of results on emotional distress.

	rhs	est	se	z	p	std.all
**Emotional distress**	Knowledge of SL	0.002	0.106	0.02	0.984	0.003
	Knowledge of SL * SA Reading Skill	−0.028	0.109	−0.258	0.796	−0.05
	Level of Deafness	0.024	0.07	0.345	0.73	0.038
	Level of Deafness * Knowledge of SL	0	0.093	−0.002	0.998	0
	Level of Deafness * Knowledge of SL * SA Reading Skill	−0.024	0.1	−0.241	0.809	−0.043
	Level of Deafness * Onset of Deafness	0.009	0.095	0.094	0.925	0.014
	Level of Deafness * Onset of Deafness * Knowledge of SL	0.023	0.052	0.439	0.661	0.038
	Level of Deafness * Onset of Deafness * SA Reading Skill	−0.087	0.09	−0.965	0.334	−0.153
	Level of Deafness * SA Reading Skill	0.084	0.058	1.458	0.145	0.149
	Onset of Deafness	−0.118	0.105	−1.119	0.263	−0.194
	Onset of Deafness * Knowledge of SL	−0.086	0.091	−0.938	0.348	−0.124
	Onset of Deafness * Knowledge of SL * SA Reading Skill	0.05	0.071	0.705	0.481	0.089
	Onset of Deafness * SA Reading Skill	0.08	0.1	0.797	0.426	0.142
	Residence	−0.035	0.086	−0.413	0.68	−0.059
	Residence* Knowledge of SL	0.051	0.096	0.532	0.595	0.09
	Residence* Knowledge of SL * SA Reading Skill	−0.055	0.098	−0.566	0.572	−0.098
	Residence * Level of Deafness	−0.083	0.058	−1.432	0.152	−0.148
	Residence * Level of Deafness * Knowledge of SL	−0.007	0.064	−0.109	0.913	−0.013
	Residence * Level of Deafness * Onset of Deafness	−0.013	0.065	−0.196	0.845	−0.023
	Residence * Level of Deafness * SA Reading Skill	−0.095	0.065	−1.472	0.141	−0.17
	Residence * Onset of Deafness	0.116	0.108	1.077	0.282	0.208
	Residence * Onset of Deafness * Knowledge of SL	−0.006	0.069	−0.09	0.929	−0.011
	Residence * Onset of Deafness * SA Reading Skill	−0.052	0.107	−0.485	0.628	−0.093
	Residence * SA Reading Skill	0.096	0.078	1.233	0.217	0.162
	SA Reading Skill	−0.121	0.082	−1.469	0.142	−0.195

**Cognitive strain** (sleep disruption, concentration difficulties): There were no significant predictors (Table 13).

**Table 13:** Summary of results on cognitive strain.

	rhs	est	se	z	p	std.all
**Cognitive strain**	Knowledge of SL	0.187	0.176	1.063	0.288	0.185
	Knowledge of SL * SA Reading Skill	0.003	0.176	0.016	0.987	0.003
	Level of Deafness	0.097	0.118	0.825	0.409	0.096
	Level of Deafness * Knowledge of SL	0.026	0.173	0.153	0.879	0.026
	Level of Deafness * Knowledge of SL * SA Reading Skill	0.033	0.182	0.182	0.856	0.036
	Level of Deafness * Onset of Deafness	−0.016	0.164	−0.1	0.921	−0.016
	Level of Deafness * Onset of Deafness * Knowledge of SL	−0.014	0.094	−0.148	0.883	−0.014
	Level of Deafness * Onset of Deafness * SA Reading Skill	−0.139	0.159	−0.874	0.382	−0.152
	Level of Deafness * SA Reading Skill	−0.034	0.102	−0.333	0.739	−0.037
	Onset of Deafness	−0.27	0.164	−1.653	0.098	−0.275
	Onset of Deafness * Knowledge of SL	0.062	0.168	0.369	0.712	0.055
	Onset of Deafness * Knowledge of SL * SA Reading Skill	0.019	0.134	0.141	0.888	0.021
	Onset of Deafness * SA Reading Skill	0.052	0.153	0.337	0.736	0.057
	Residence	−0.077	0.132	−0.581	0.561	−0.079
	Residence* Knowledge of SL	0.141	0.201	0.705	0.481	0.154
	Residence* Knowledge of SL * SA Reading Skill	−0.172	0.194	−0.885	0.376	−0.189
	Residence * Level of Deafness	−0.06	0.1	−0.606	0.544	−0.066
	Residence * Level of Deafness * Knowledge of SL	−0.064	0.115	−0.553	0.58	−0.07
	Residence * Level of Deafness * Onset of Deafness	0	0.117	0.002	0.999	0
	Residence * Level of Deafness * SA Reading Skill	−0.113	0.113	−0.998	0.318	−0.124
	Residence * Onset of Deafness	0.203	0.21	0.962	0.336	0.224
	Residence * Onset of Deafness * Knowledge of SL	−0.083	0.109	−0.759	0.448	−0.09
	Residence * Onset of Deafness * SA Reading Skill	0.061	0.205	0.297	0.766	0.067
	Residence * SA Reading Skill	0.076	0.103	0.737	0.461	0.078
	SA Reading Skill	−0.006	0.148	−0.039	0.969	−0.006

**Sadness and loneliness:** There was a significant three-way interaction between Residence, Level of Deafness, and SA Reading Skill, *b* = −0.24, *SE* = 0.11, *z* = −2.18, *p* = .029. All other main effects and interactions were not significant (all *ps* ≥ .111). Simple-effect analyses of the interaction showed that UK HoH skilled readers reported higher scores than their Spanish HoH skilled readers (*b* = 0.86, *SE* = 0.35, *z* = 2.45, *p* = .014), and Deaf Spanish skilled readers reported higher scores than HoH Spanish participants (*b* = 0.91, *SE* = 0.40, *z* = 2.25, *p* = .025). A marginal trend suggested that in Spain HoH skilled readers scored lower than HoH less-skilled readers (*b* = −1.05, *SE* = 0.55, *z* = −1.91, *p* = .056). All other simple effects were non-significant (all *ps* ≥ .290) (Table 14).

**Table 14:** Summary of results on sadness and loneliness.

	rhs	est	se	z	p	std.all
**Sadness and loneliness**	Knowledge of SL	0.1	0.176	0.571	0.568	0.096
	Knowledge of SL * SA Reading Skill	−0.048	0.176	−0.275	0.784	−0.051
	Level of Deafness	0.056	0.125	0.451	0.652	0.054
	Level of Deafness * Knowledge of SL	−0.083	0.115	−0.723	0.47	−0.08
	Level of Deafness * Knowledge of SL * SA Reading Skill	0.037	0.146	0.255	0.798	0.04
	Level of Deafness * Onset of Deafness	0.098	0.117	0.845	0.398	0.094
	Level of Deafness * Onset of Deafness * Knowledge of SL	−0.06	0.097	−0.619	0.536	−0.059
	Level of Deafness * Onset of Deafness * SA Reading Skill	−0.11	0.124	−0.891	0.373	−0.116
	Level of Deafness * SA Reading Skill	0.14	0.104	1.348	0.178	0.148
	Onset of Deafness	−0.142	0.134	−1.06	0.289	−0.14
	Onset of Deafness * Knowledge of SL	0.119	0.172	0.69	0.49	0.102
	Onset of Deafness * Knowledge of SL * SA Reading Skill	−0.115	0.146	−0.788	0.43	−0.122
	Onset of Deafness * SA Reading Skill	0.056	0.129	0.432	0.666	0.06
	Residence	0.038	0.158	0.242	0.809	0.038
	Residence* Knowledge of SL	0.195	0.123	1.587	0.112	0.205
	Residence* Knowledge of SL * SA Reading Skill	−0.204	0.128	−1.592	0.111	−0.217
	Residence * Level of Deafness	−0.021	0.101	−0.212	0.832	−0.023
	Residence * Level of Deafness * Knowledge of SL	−0.058	0.116	−0.496	0.62	−0.061
	Residence * Level of Deafness * Onset of Deafness	−0.083	0.109	−0.765	0.444	−0.089
	Residence * Level of Deafness * SA Reading Skill	**−0.238**	**0.109**	**−2.178**	**0.029**	**−0.254**
	Residence * Onset of Deafness	−0.01	0.163	−0.059	0.953	−0.01
	Residence * Onset of Deafness * Knowledge of SL	−0.026	0.12	−0.218	0.827	−0.028
	Residence * Onset of Deafness * SA Reading Skill	0.147	0.167	0.879	0.379	0.157
	Residence * SA Reading Skill	0.133	0.144	0.924	0.356	0.133
	SA Reading Skill	−0.013	0.132	−0.101	0.919	−0.013

**Less active:** There were no significant effect (all p values > .05) (Table 15).

**Table 15:** Summary of results on reduced activity.

	rhs	est	se	z	p	std.all
**Less active**	Knowledge of SL	0.156	0.288	0.543	0.587	0.126
	Knowledge of SL * SA Reading Skill	−0.092	0.255	−0.36	0.719	−0.083
	Level of Deafness	−0.161	0.208	−0.772	0.44	−0.13
	Level of Deafness * Knowledge of SL	−0.254	0.351	−0.722	0.47	−0.206
	Level of Deafness * Knowledge of SL * SA Reading Skill	0.254	0.303	0.838	0.402	0.228
	Level of Deafness * Onset of Deafness	0.288	0.232	1.243	0.214	0.232
	Level of Deafness * Onset of Deafness * Knowledge of SL	−0.058	0.109	−0.532	0.595	−0.048
	Level of Deafness * Onset of Deafness * SA Reading Skill	−0.406	0.251	−1.618	0.106	−0.363
	Level of Deafness * SA Reading Skill	0.139	0.141	0.986	0.324	0.125
	Onset of Deafness	−0.406	0.21	−1.93	0.054	−0.339
	Onset of Deafness * Knowledge of SL	0.052	0.328	0.158	0.874	0.038
	Onset of Deafness * Knowledge of SL * SA Reading Skill	−0.185	0.29	−0.636	0.525	−0.165
	Onset of Deafness * SA Reading Skill	0.118	0.184	0.642	0.521	0.107
	Residence	0.005	0.206	0.027	0.979	0.005
	Residence* Knowledge of SL	0.26	0.316	0.823	0.411	0.232
	Residence* Knowledge of SL * SA Reading Skill	−0.351	0.313	−1.123	0.261	−0.316
	Residence * Level of Deafness	0.023	0.269	0.086	0.931	0.021
	Residence * Level of Deafness * Knowledge of SL	−0.08	0.155	−0.517	0.605	−0.072
	Residence * Level of Deafness * Onset of Deafness	0.099	0.146	0.675	0.5	0.089
	Residence * Level of Deafness * SA Reading Skill	−0.062	0.203	−0.304	0.761	−0.056
	Residence * Onset of Deafness	−0.23	0.366	−0.629	0.529	−0.209
	Residence * Onset of Deafness * Knowledge of SL	−0.011	0.148	−0.075	0.94	−0.01
	Residence * Onset of Deafness * SA Reading Skill	0.272	0.336	0.807	0.42	0.246
	Residence * SA Reading Skill	−0.009	0.16	−0.059	0.953	−0.008
	SA Reading Skill	0.151	0.294	0.513	0.608	0.123

## Discussion

Accessible messages are fundamental for effective communication of public health information to the whole of society. The present work reports the experiences with accessibility of information and mental health of a large sample of deaf/HoH people in the UK and Spain during the COVID-19 pandemic. But note that although the COVID-19 pandemic created a unique context for this study, we study communication barriers faced by deaf people in their daily lives. Hence, our findings go beyond accessibility during a global health emergency. We used an accessible survey, distributed in written English and Spanish as well as in three different SL (BSL, LSC and LSE), that captured responses from 285 signers. This allowed for representation of deaf signers with little access to written language who are likely to face the strongest communication barriers (for rationale and details see discussion in [1]. We propose that the high number of deaf people completing the signed version of our survey highlights the importance of dedicating more resources (i.e., funds and time necessary to create adequate SL clips) so sign language is used as a main language in research involving deaf people.

We found that most deaf/HoH people relied on subtitles to access information from the government and the news. This is not surprising given the low rate of SL interpreting available. Bosch-Baliarda et al. [52] reported that across the European Broadcasting Union in 2016 only 4% of the programmes were delivered in SL, to the best of our knowledge this proportion did not increase during the COVID-19 pandemic. As a consequence, deaf people’s only ways to access televised materials were a combination of residual hearing, lipreading and subtitle reading. Our findings are consistent with previous research showing deaf readers have a positive attitude towards reading well-timed, informative subtitles and read subtitles frequently [53], but often experience difficulties accessing the information that result on more dwell time and more fixations compared to hearing viewers [53–57]. Indeed, age and reading skill seem to play a large role on difficulty of subtitle reading in deaf people [55]. Written text was the modality in which deaf/HoH people found most of the information in social media and websites. Reading subtitles and texts available online to stay informed is likely to have driven the increase in the number of hours that 40% of deaf/HoH people read during lockdown periods, increase that was not related to more availability of books [18].

Finally, SL was the chosen modality to access information from Deaf organisations. Despite the efforts to keep the population informed, most deaf people found accessible only ‘some of the time’ or less frequently. Similarly, most deaf people were less than moderately satisfied with the information they received, particularly with information from the government, social media and websites. This is consistent with findings that deaf patients report lower satisfaction with health care quality and access than other patients [58], particularly of those without SL support for communication with health care providers. Reeves et al. [59] found that even when 50% of deaf users requested SL interpretation, interpreters were present at only 17% of doctor’s appointments and there was even less availability (7%) at accidents and emergencies consultations. This low availability of communication via SL resulted on lower satisfaction levels with the health care system. It is clear in our data that deaf people relied on the sign language content whenever it was available (e.g., in small proportion for the government, but in a larger proportion from deaf organisations) which supports the case for increasing availability of SL content across all possible sources of information.

Furthermore, physical and most dimensions of mental wellbeing appeared to decline during the pandemic. Because SF-12 has not been validated for deaf/HoH populations, we do not interpret these scores clinically or diagnostically, all results reported here reflect trends and associations within tis deaf/HoH sample. Physical health for our participants suggested average physical health in relation to pre-pandemic averages of the general population. We found differences in physical health related to Onset of Deafness, and to the interaction between Reading Skill and Knowledge of SL. Specifically, late onset deaf/HoH people had worst physical health than early onset deaf/HoH people, among signers skilled readers had better physical health than less skilled readers, and amongst less skilled readers non-signers had better physical health than signers. This is an important outcome of this research and it offers further insight to previous research showing that deaf people’s health is lower than their hearing counterparts [60,61] and they face higher risks of injury, death, and property loss as documented in recent U.S. disasters [25,62,63]. Importantly, signers were at disadvantage if less-skilled readers. The overall SF-12 mental health scores falling below the typical population’s average may be attributed to the broader impact of the COVID-19 pandemic—likely affecting many hearing communities as well. Interestingly, late-onset deaf people and signers report lower mental health scores compared to those with early-onset deafness and non-signers, respectively. This latest finding is not consistent with signers having better support networks than their counterparts. As in Gutierrez-Sigut [1], it is clear from the present findings that a one‑size‑fits‑all approach will not be effective to decrease communication barriers for deaf/HoH people. More research is needed to disentangle the complex interaction between different aspects of deafness. The remainder of this section will discuss the distinct impact of our predictor variables, focusing on reading ability, which emerged as a crucial factor for accessibility.

### Reading skill

Reading skill was the most pervasive and influential factor in determining both the quantity and quality of information access and satisfaction across most sources. Reading skill was a main predictor of accessibility of information necessary to stay safe and accessibility and satisfaction with information from official sources. Reading skill interacted with Knowledge of SL to explain physical health. Reading skill was also involved in exploratory three-way interactions; for example, it interacted with Onset of Deafness and Knowledge of Sign Language to explain both accessibility and satisfaction with information from deaf organizations, with residence and Knowledge of SL to predict satisfaction with information from deaf organizations, and with residence and level of deafness to predict the sadness and loneliness aspect of mental health.

Across the board, less-skilled readers were at disadvantage for access to information than skilled readers, with this effect evident in general safety information, governmental(official) sources accessibility and satisfaction, and potentially playing a role in accessibility and satisfaction with information from deaf organisations. Reading skill interacted with Knowledge of SL, Onset of Deafness, and Residence, revealing that higher reading skill likely functions as a protective resource, buffering against other potential barriers, for deaf/HoH adults’ access to enough information to stay safe and is a main predictor of access and satisfaction with official sources. Conversely, lower reading skill particularly disadvantages signers although late-onset deaf/HoH people with lower reading skill were also at disadvantage in their ability to access enough information to stay safe and were less satisfied with information from official sources. These findings are not only not surprising but also consistent with findings from the general hearing population. Several studies have shown that even when the quality of online information provided is fair, its readability often surpasses the recommended levels and this limited accessibility for many users [64–66]. It is well know that when the communication methods do not match recipients’ abilities, users are not able to process and understand health information that leads them to appropriate health decisions.

In the present study reading skill predicted deaf/HoH people’s physical health raw scores, with no differences depending on Knowledge of SL for skilled readers but poorer health in signers if they were low skilled readers. Based on an estimate of only ten percent of the deaf population in the USA being both proficient signers and skilled readers, it has been recommended that communication for Deaf populations should be at nine to ten years old level or below, and include ASL resources developed with Deaf/HH users’ input (for a recent review see [18]. The present study highlights the importance of following this recommendation, showing the negative impact of high reading level written information combined with general lack of SL interpretation on accessibility, satisfaction and mental health.

We did not capture worse mental health outcomes in less-skilled readers after aspects of mental health were combined via factor analyses. This contrasts with findings in the general population showing that reading difficulties are related to poorer mental health outcomes [67,68]. Previous studies with deaf people have shown a correlation between language skills and mental health [69–71] although those studies have focused on SL ability to assess language skill. For example, Øhre et al. [71] observed that adult deaf/HoH spoken language users had higher levels of stress than those who used sign language. The fact that we do not find strong impact of reading skill in mental health can be due to the combination of slightly different aspects into a more general factor that loses some specificity needed for this population.

Finally, in the present study we addressed reading news to keep up with current events, which has been identified by several large studies as one of five main reading activities common in adulthood together with reading for leisure, reading study or work documents, reading to socialize with others, and shared reading with children (see [72–74]. All of these reading habits have been shown to be linked with psychological, emotional, and health conditions in hearing people (for review see [75]. We expect that similar patterns to what we found in the present study are present for deaf readers in all reading habits.

### Onset of deafness

Onset of Deafness showed modest but consistent effects. Onset of Deafness was a main predictor of physical and mental health, and it was a moderator of general access to enough information to stay safe and satisfaction, interacting with reading skill, Level of Deafness, Knowledge of SL, and Residence. HoH people who became deaf after they were 15 years old (late-onset) and were skilled readers had higher accessibility than low-skilled readers. Late-onset deaf/HoH low skilled readers were less satisfied with official sources of information in the UK than in Spain, but even in Spain late-onset lower skill readers were less satisfied than skilled readers. These findings could reflect that late onset deaf people acquired reading before becoming deaf and hence were more likely to have a higher reading ability than early onset reading. This interpretation is supported by a significant albeit weak correlation between reading skill and Onset of Deafness (*R* = −.132, *p* < .01). People with later-onset deafness were on average older than the early onset deafness respondents and likely they had established newspaper-reading routines, while the younger demographic tends to be more inclined towards digital media (see, e.g., [76]. It is well-known that as people age, they often start reading newspapers more frequently (e.g., [77–82]. This is in part because older people usually have more time to read, while younger people report that they do not have the time required and that they find reading newspapers inconvenient [83]. Interestingly, information from deaf organisations was more accessible for late onset less skilled readers who sign than those that do not sign. Reliable information provided in an accessible format by deaf organisations is important for a group as vulnerable as early onset signers with low reading ability, who might not access information from any other source. As an additional benefit, information being provided by deaf organisations can also benefit late-onset with lower reading skill. It is well known that those with late onset deafness usually report lack of belonging to both the hearing and the signing world [84,85]. Having socialised as hearing people until they became deaf, they struggle to adapt to non-hearing dependent modes of communication [86]. Research indicates that late-onset deaf adults have a higher likelihood of divorce compared to their hearing counterparts [87,88], and often feel isolated within their own families due to communication barriers [85]. Social interactions are further limited by the difficulty of engaging in conversations with multiple people amidst background noise [89]. Many late onset deaf people never establish effective communication strategies, leading to significant strain in their relationships [90,91]. Although confirmatory evidence is needed, our findings suggest that learning to sign and engaging with information provided by deaf organisations can have a positive effect.

Crucially, in this sample both physical and mental health were poorer among those with late-onset deafness. We think that these differences are likely due to their older age and the lack of a social network that uses visual communication as a main vehicle, and the perception of deafness as a disability rather than as part of naturally occurring diversity. We have previously found [1] that late onset deaf people experienced more difficulties in communicating than early onset deaf people when their interlocutors wore masks. We argued that because of their shorter experience with deafness, people with late onset deafness are less likely than early onset deaf people to have developed communication strategies involving a wider range of visual information. We also argued that people who became deaf later in life could be more affected by the feelings of shame and the stigma associated with deafness perceived as a disability or a loss of a function [92]. Deaf/HoH people who belong to a deaf community (e.g., early-onset deaf people immersed in deaf culture), are likely to consider deafness as an integral part of their identity and be less influenced by stigma [93,94]. We propose that these factors are more likely to explain the lower mental health scores for late-onset deaf/HoH people found here than their ability to access information from multiple sources.

### Level of deafness

Level of Deafness had a minimal effect across outcomes, influencing access and satisfaction in nuanced ways and in interaction with reading skill and Onset of Deafness. For example, HoH participants with late onset who were skilled readers reported significantly higher accessibility than less-skilled counterparts.

### Knowledge of SL

Knowledge of SL was a main predictor of general accessibility of information to stay safe, satisfaction with information from official sources, and both physical and mental health, although often interacted with reading skill. In general signers reported less access to enough information to stay safe than non-signers particularly among less-skilled readers, as among skilled readers SL knowledge had no effect. Accessibility to official sources was poorer for signers than non-signers specially in the UK, but not in Spain where a larger proportion of TV news were interpreted to SL more consistently than in the UK.

Similarly, signers reported substantially poorer physical health than non-signers, particularly those with low reading skills as this gap disappeared among skilled readers. For mental health, signers reported worse mental health than non-signers. SL knowledge combined with Onset of Deafness and reading skill predicted satisfaction with information from deaf organisations, favouring skilled signers with early onset. Importantly, SL was the preferred way to access information from deaf organisations, with well over one hundred and fifty respondents making use of these resources. Indeed, signers reported more frequent use of sign language to access information from deaf organisations. In the same way that access to health information in SL is crucial for deaf signers (for a review see [95,96], we believe that these signed updates were of vital importance for signers and accomplished a necessary social function without which accessibility and satisfaction levels could have been much lower.

### Socio-cultural context

Country of residence had no significant main effect on physical or mental health but emerged as a strong main predictor of general accessibility and accessibility of information from community sources and satisfaction with official sources; with UK participants reporting higher general accessibility to safety information and higher satisfaction with official sources, but lower accessibility from the community sources than Spanish participants. Regarding potential complex interactions, the influence of the country of residence was moderated by Reading Skill, Knowledge of SL, and Onset of Deafness. Interaction trends suggested that signers were at disadvantage accessing information from official sources in the UK but not in Spain. Furthermore, satisfaction with official sources of information was higher in Spain than in the UK for late-onset deaf/HH people with lower reading skill. This is likely to be due to more availability of interpreted TV and media news reports in Spain than in the UK.

Crucially, access to community sources was higher in Spain than in the UK. This might reflect that Spanish HoH individuals relied more on community and family as an information source during the pandemic compared to their UK counterparts. Comparative research shows variation depending on cultural context of family structure and people’s understanding of familiar duties [96]. In relation to the UK and northern Europe, family organization in southern Europe shows more inter-generational dependence, stronger kinship ties, greater responsibility for the welfare of the family members, and higher interrelation between family and economic structure (e.g., there is more proliferation of family businesses) (for a review see [97]. In the north young people leave home earlier and not necessarily to form a nuclear family of their own, while in the south people delay the age they leave their parents’ home [98]. In addition, people in Spain are generally more collectivistic [99], and hence are part or stronger and more cohesive networks [100]. Being closer to family and friends might have facilitated communication and access to information from them.

## Limitations

We focused on accessibility and were able to achieve a better representation of the deaf population, including deaf/HoH people whose first language is one of the three studied sign languages. This addressed limitations of previous research and included signers that have little access to written language (i.e., lower reading skill). However, we acknowledge that the online survey excluded those with lower computer literacy or poor internet access. This group may face greater impacts on health due to limited information access. Future research should address this by conducting face-to-face interviews in their preferred communication method. Future research should also be targeted towards a larger and more diverse group of deaf people in different countries. In the present study, we included a large number of theoretically relevant predictors. However, the sample size and uneven subgroup distributions mean that finding from three‑way interactions are exploratory and require confirmation in studies designed to test them. Given the relevance of Onset of Deafness, and Knowledge of SL further research recruiting a larger number of participants would benefit from breaking down those factors, for example studying early and late-onset deafness separately and distinguishing native signers from those who learned sign language at school. It would also be desirable to consider SL proficiency as a predictor of accessibility and satisfaction with information in future research with signers. It would be advisable for future research to collect data in a way that allows for evaluating the effectiveness of distribution channels. Finally, our mental health questions referred to negative emotional experiences, we acknowledge that this might not include a balanced view of mental health during the pandemic. Future research should also include questions aimed to capture any positive change as well as the range of coping strategies.

## Conclusions

We studied access to information of deaf people, the COVID-19 pandemic created a unique context for this study, but our study reflects ongoing communication issues.

We learnt that signed information was preferred by a large percentage of deaf people when available (e.g., from deaf organisations with 49% satisfied with information from deaf associations compared to 26% from government TV updates and 22% from websites. This supports the need to expand access to information in sign language by increasing the availability of qualified interpreters, enhancing the provision of interpreted content through public channels, and supporting additional signed initiatives led by deaf associations.

Since information in SL was scarce, most deaf/HoH people relied on subtitles to access information from the government, news and websites. Given this, it is not surprising that reading ability emerged as a key factor related to access, satisfaction, and health. Our findings advocate for releasing written information at appropriate reading levels as well as for improving literacy programmes for deaf people. Increasing access to written information, either by simplifying reading levels or improving reading skills, can mitigate the negative health consequences of limited information access. Community support is vital for low-skilled readers.

Our findings align with well-documented systemic barriers, particularly the persistent lack of access to qualified interpreters and the over-reliance on written or spoken language, which often marginalizes individuals who primarily communicate through sign language [26–33]. These results highlight the urgent need for targeted interventions aimed at reducing these barriers and ensuring equitable access to information and services for the deaf and hard-of-hearing community. Previous studies in the area of education have advocated for promoting self-determination and empowerment for Deaf/HoH individuals via training in self-advocacy strategies, increasing educators awareness of deafness, encouraging self-regulation during learning tasks, and above all providing accessible communication using subtitles and sign language interpreters [101–109]. We believe that interventions based on those strategies can succeed to improve accessibility to general health and social communication as well.

The datasets generated and analysed during the current study, as well as the full regression analyses results, are available in the OSF repository https://osf.io/gzdt9/?viewonly=abc0e87dacea42978f21973370eaf0c8

We would like to thank the Royal Association for Deaf people (RAD) for their collaboration and support. We would also like to thank all the deaf respondents that gave up their time.
